# Diagnosis of pregnancy disorder in the first‐trimester patient plasma with Raman spectroscopy and protein analysis

**DOI:** 10.1002/btm2.10691

**Published:** 2024-07-16

**Authors:** Ansuja P. Mathew, Gabriel Cutshaw, Olivia Appel, Meghan Funk, Lilly Synan, Joshua Waite, Saman Ghazvini, Xiaona Wen, Soumik Sarkar, Mark Santillan, Donna Santillan, Rizia Bardhan

**Affiliations:** ^1^ Department of Chemical and Biological Engineering Iowa State University Ames Iowa USA; ^2^ Nanovaccine Institute Iowa State University Ames Iowa USA; ^3^ Department of Obstetrics and Gynecology, Carver College of Medicine University of Iowa Hospitals & Clinics Iowa City Iowa USA; ^4^ Department of Mechanical Engineering Iowa State University Ames Iowa USA

**Keywords:** first trimester, gestational diabetes, mass spectrometry, metabolism, pregnancy, Raman spectroscopy

## Abstract

Gestational diabetes mellitus (GDM) is a pregnancy disorder associated with short‐ and long‐term adverse outcomes in both mothers and infants. The current clinical test of blood glucose levels late in the second trimester is inadequate for early detection of GDM. Here we show the utility of Raman spectroscopy (RS) for rapid and highly sensitive maternal metabolome screening for GDM in the first trimester. Key metabolites, including phospholipids, carbohydrates, and major amino acids, were identified with RS and validated with mass spectrometry, enabling insights into associated metabolic pathway enrichment. Using classical machine learning (ML) approaches, we showed the performance of the RS metabolic model (cross‐validation AUC 0.97) surpassed that achieved with patients' clinical data alone (cross‐validation AUC 0.59) or prior studies with single biomarkers. Further, we analyzed novel proteins and identified fetuin‐A as a promising candidate for early GDM prediction. A correlation analysis showed a moderate to strong correlation between multiple metabolites and proteins, suggesting a combined protein‐metabolic analysis integrated with ML would enable a powerful screening platform for first trimester diagnosis. Our study underscores RS metabolic profiling as a cost‐effective tool that can be integrated into the current clinical workflow for accurate risk stratification of GDM and to improve both maternal and neonatal outcomes.

**Translational Impact Statement**

The utility of Raman spectroscopy (RS) as a clinically relevant tool is demonstrated for early screening of gestational diabetes during the first trimester. By metabolic profiling of patient plasma and combining RS data with machine learning (ML), we achieved an unprecedented accuracy of >90%, surpassing current clinical tests and prediction models. In addition to metabolites, we also identified new protein markers, and showed correlations of metabolites to proteins, and to patient clinical factors. Our findings suggest RS combined with protein analysis and ML may enable a transformative shift in the early and rapid detection of pregnancy disorders.

## 1 INTRODUCTION

Gestational diabetes mellitus (GDM), characterized by the spontaneous development of hyperglycemia during pregnancy, impacts ~14% of pregnancies globally.^1^ GDM is exaggerated by risk factors including obesity, familial diabetes, advanced maternal age, and nutrient‐deficient diet.^2^ Population‐wide studies report a steady increase in GDM cases annually in the United States. Further, there are significant racial and ethnic disparities in both the occurrence of GDM, and the resulting adverse outcomes that include preterm birth, small or large neonate for gestational age, and gestational hypertension.^3^, ^4^ Whereas lifestyle interventions such as diet and exercise, insulin therapy, and oral hypoglycemic medications are widely used to manage GDM, they have not been effective in mitigating maternal and neonatal risks.^1^, ^5^, ^6^ The physiological risks of GDM extend beyond the gestational period and persist into later life for both mother and children, leading to morbidities such as type 2 diabetes, obesity, and metabolic syndrome.^6^, ^7^, ^8^ In the United States, the current clinical approach screens for GDM in the late second trimester (24–28 weeks); to be ruled out patients must meet glycemic targets of fasting plasma glucose <92 mg/dL and either a 1‐h glucose threshold <180 mg/dL or a 2‐h threshold <153 mg/dL set by the International Association of Diabetes and Pregnancy Study Groups.^9^ But this approach is inadequate in predicting those at risk of developing GDM early in the pregnancy. Many risk‐scoring systems have been established based on a patient's age, body mass index (BMI), and family history of diabetes. However, the accuracy of these models has been ≤60% and only improved slightly when the history of GDM in prior pregnancies was included.^10^, ^11^ Therefore, current prediction models are often inconclusive and may not be applicable to nulliparous women.

Advances in omics approaches, including metabolomics, have led to a paradigm shift in the early diagnosis of many disorders. Since GDM reflects an extreme manifestation of metabolic alterations, recent findings have leveraged metabolomics to identify key metabolites contributing to the pathophysiology of GDM.^12^ Yet, the majority of these clinical studies have focused on late second and third trimester metabolites^13^, ^14^, ^15^ and a few in the early second trimester.^16^, ^17^ Metabolic assessment in the first trimester remains underdeveloped. This knowledge gap may be in part because few metabolites are enriched in maternal circulation very early in the pregnancy, falling below the sensitivity level of current gold standard techniques (such as nuclear magnetic resonance and mass spectrometry [MS]). Further, metabolites are also extremely sensitive to their environment, where complex metabolite extraction processes in poorly enriched samples can often lead to measurement inconsistencies. MS approaches are also often limited by matrix effects, where the ionization of target analytes is altered, that leads to signal suppression.^18^, ^19^ Finally, while expansive metabolomics databases are available for metabolite identification, confounding overlap with unrelated molecules can often influence the results. Raman spectroscopy (RS) has emerged as a powerful optical technique offering a label‐free, extraction‐free, low cost, and rapid approach for metabolic profiling in cells, tissues, and patient biofluids.^20^, ^21^ RS measures the inelastic scattering of incident light via the molecular vibrations of the various biochemical species in a sample.^22^ The unique spectra resulting from this light scattering include many classes of metabolites such as nucleic acids, amino acids, fatty acids, and sugars among others with well‐established peak positions.^23^ Indeed RS has enabled early detection and treatment monitoring in multiple disease models by identifying specific spectral biomarkers. These include the identification of tumor margin for resection,^24^, ^25^ distinguishing various pathogen strains and assessing antimicrobial susceptibility,^26^, ^27^ diagnosis of metabolic disorders by allowing a non‐invasive alternative for glucose monitoring,^28^, ^29^, ^30^ and identification of inflammatory processes and tissue damage characteristic of gut disorders.^31^, ^32^ Our group and others have shown that RS is highly sensitive, even at low metabolite concentrations in the early diagnosis and prognosis of metabolically active diseases such as cancer.^33^, ^34^, ^35^ This work also builds upon our prior studies in pregnancy disorders, in which RS data combined with clinical information were used to predict preterm birth in the first trimester^36^ and for the longitudinal screening of the maternal metabolome of preeclamptic patients.^37^

In this work, the strengths of RS were leveraged for maternal metabolome screening in first‐trimester pregnant patient plasma who were later diagnosed with GDM and compared to those who had normal blood sugar levels throughout pregnancy (healthy). Raman spectral results were integrated with unsupervised machine learning (ML) for data visualization to distinguish the two patient cohorts. We identified key metabolites that were correlated with the late second trimester blood sugar levels. Raman findings were validated with MS metabolomics showing synergy between the two approaches in metabolites measured, and the related metabolic pathways enriched were identified using the Kyoto Encyclopedia of Genes and Genomes (KEGG) database. We leveraged a support vector machine (SVM) model and achieved an area under the curve (AUC) of 0.59 ± 0.12 for the clinical data alone, that includes demographics and obstetric data shown in Table 1. The Raman metabolites achieved an AUC of 0.97 ± 0.06, highlighting the ability of RS to make highly accurate predictions even with a small sample size. Protein analysis with an enzyme‐linked immunosorbent assay (ELISA) identified fetuin‐A as a promising marker that would complement metabolic profiling early in pregnancy. A correlation analysis of metabolites to clinical data and metabolites to proteins showed moderate to strong correlation, suggesting that a combined metabolic and protein screening may enable GDM risk stratification. We demonstrate that RS has the potential to complement the current clinical workflow for early assessment of the risk of pregnancy disorders.

**TABLE 1:** Average demographic, obstetric history, and clinical information of patients.

Clinical parameter of patients	Healthy (mean ± SD)	GDM (mean ± SD)	*p*‐Value
Number of patients	34	34	–
Maternal age (years)	31.8 ± 4.7	31.9 ± 5.6	0.463
BMI (kg/m^2^)	29.5 ± 10.1	31.9 ± 7.8	0.138
Gestational age (weeks)	38.5 ± 1.8	38.8 ± 1.3	0.281
Gravida	2.5 ± 1.5	2.7 ± 1.7	0.306
Parity	1.1 ± 0.9	1.2 ± 1.4	0.46
Pregnancy loss	0.4 ± 0.7	0.5 ± 0.8	0.322
Blood sugar concentration (mg/dL)	116.0 ± 20.1	185.4 ± 29.2	1.07E−16
Comorbidities (%)	17.6	2.9	0.0234

Abbreviations: BMI, body mass index; GDM, gestational diabetes mellitus.

## 2 RESULTS AND DISCUSSIONS

In this study, *n* = 34 first‐trimester plasma samples of pregnant patients who were diagnosed with GDM later in the pregnancy were evaluated and compared to *n* = 34 patients with healthy pregnancies. This research was reviewed and approved by the Institutional Review Board (IRB#200910784). The coded samples were received from the Perinatal Family Tissue Bank (PFTB) at the University of Iowa (UI), which is an academic biobank where patient biofluids are continuously banked for research and education purposes from patients who provided informed consent. Patients were not specifically recruited for this study but had previously consented to have their samples stored and made available for research purposes. First‐trimester samples are in high demand at this tissue bank, and thus we were limited in the number of samples that were available to us. Therefore, for the healthy patients' data, we used our group's previously published Raman data set^37^ that was released to the Iowa State University's open data repository^38^ and supplemented with additional healthy patient samples. It is also important to note that the majority of our subjects are White (85%), which is representative of the Iowa population. Table 1 summarizes the average maternal demographic data, including maternal age, BMI, number of pregnancies (gravida), number of births (parity), pregnancy losses, gestational age at delivery, blood sugar from a glucose test taken after 24 weeks, and % comorbidities. This information for individual de‐identified patients in the two cohorts is provided in Tables S1 and S2. The average 1‐h blood glucose level for the GDM patients was 185.4 ± 29.2 mg/dL, which was significantly higher than that of the healthy patients.

**FIGURE 1:**
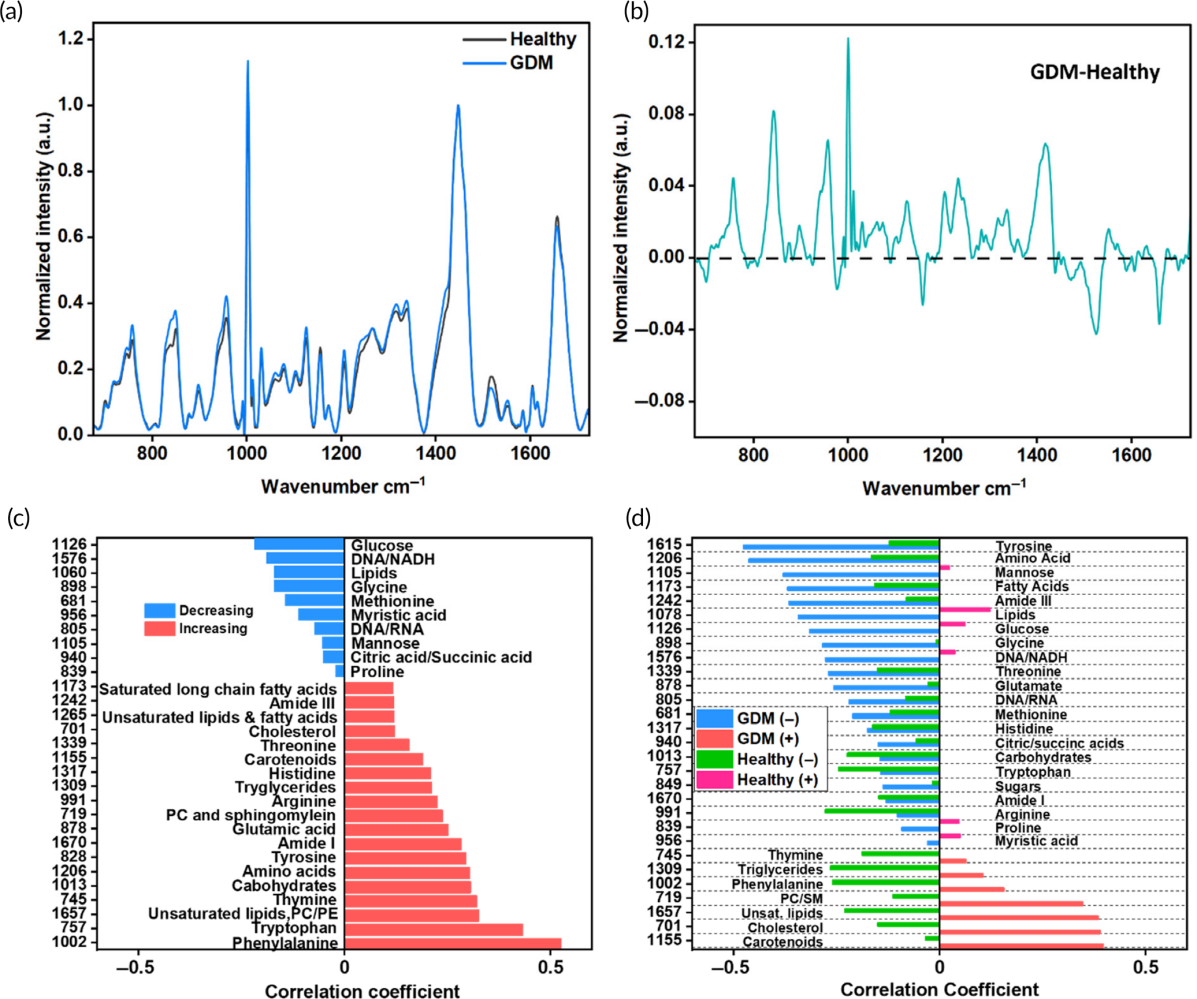
Raman spectroscopy (RS) can distinguish healthy pregnancy and pre‐gestational diabetes mellitus (GDM) patients. (a) Representative Raman spectra for healthy and GDM samples. (b) Difference spectra for the spectra in (a). (c) Pearson's correlation analysis of significant metabolites to GDM status using *n* = 34 GDM patient samples and *n* = 34 healthy patient samples. PC, phosphatidylcholine; PE, phosphatidylethanolamine; SM, sphingomyelin. (d) Pearson correlation between first trimester plasma RS metabolites and late second trimester blood glucose levels for both *n* = 34 GDM patient samples and *n* = 34 healthy patient samples. (+) increasing, (−) decreasing.

The Raman spectra of the GDM and healthy patient samples were obtained by measurement with a 785 nm laser with 40 mW power at the point of excitation using a 50× objective. The measured spectra were smoothed, background corrected, normalized with the standard normal variate (SNV) method, and then ratiometrically analyzed using the 1448 cm^−1^ lipid and protein‐associated peak. This peak was chosen because of its negligible variation among the samples. Complete RS methods can be seen in the experimental methods section and Figure S1. The Raman spectra of healthy and GDM patients were first compared to identify changes in their metabolic profiles. Normalized representative Raman spectra of a healthy and a GDM patient are shown in Figure 1a, and the normalized Raman spectra of all healthy and GDM patients are shown in Figure S2. The differences in the RS profiles between the two cohorts (Figure 1b) highlight metabolic changes at the onset of disease. The difference spectrum was obtained by subtracting the healthy spectrum from the GDM spectrum; hence, the positive values identify increases in abundance of associated metabolites, and negative values represent a decrease in metabolites. Tentative Raman peaks are then assigned to the corresponding metabolites based on previous studies from our group,^36^, ^37^ as well as highly cited and established Raman references on biological samples such as tissues, blood, and blood components noted in the reference list of Table 2. We also reviewed the literature based on metabolomic studies in pregnancy disorders to confirm the relevance of the identified metabolites in GDM.^46^, ^47^ The detailed list of Raman peaks, their tentative metabolic assignments, and the corresponding vibrational modes are listed in Table 2.

**TABLE 2:** Tentative peak assignments for Raman spectral data including the vibrational modes.

Wavenumber (cm^−1^)	Metabolic assignment	Vibrational modes	References
681	Methionine	CO_2_‐wagging	39
701	Cholesterol	Choline group, CH_2_ rocking	41, 42, 43
719	Phosphatidylcholine (PC)/sphingomyelin	Symmetric stretch vibration of choline group	41, 43
745	Thymine (DNA bases)	Backbone vibrations, deformation of the ring	40, 41
757	Tryptophan	Symmetric breathing of tryptophan, σ(ring)	39, 40, 41
788	DNA/RNA	C'–O–P–O–C'3 phosphodiester bonds	41, 44
805	DNA/RNA	C'–O–P–O–C'3 phosphodiester bonds	40, 41, 44
828	Tyrosine	Ring breathing	39, 40, 41
839	Proline	C–C stretching	39, 40
849	Sugars (glucose and glycerol)	C–O–C skeletal stretching	40, 41
878	Glutamic acid	C–O–C ring, C–O–H bending	39, 40
898	Glycine	C–C stretching, O–C–O stretching	39, 40
940	Citric acid, succinic acid	υ (OH⋯O) out of plane wagging vibration of hydrogen bonds	40
956	Myristic acid	C–H bending	40, 42
991	Arginine	C–N stretching	39, 40
1002	Phenylalanine	Symmetric ring breathing	41
1013	Carbohydrates	C–O–C ring, C–O–H bending	41, 45
1030	Phenylalanine	C–H in‐plane bending	39, 40, 41
1060	Lipids	Chain C–C stretching	40, 41, 42
1078	Lipids	Chain C–C stretching	40, 41, 42
1105	Mannose	σ(CH_2_) twisting vibrations	40, 41, 44
1126	Glucose	C–C stretching, C–O stretching, C–O–H in‐plane bending	40, 41, 45
1155	Carotenoids	C–C stretching and C–H stretching	40, 41, 43
1173	Saturated fatty acids	C–C stretching (skeletal option)	40, 41, 42
1206	Amino acids	NH_3_ asymmetric rocking	39, 40
1242	Amide III	Asymmetric O–P–O stretching	39, 41, 43
1265	Unsaturated lipids, fatty acids	=CH deformation, C–H stretching	41, 42, 43
1309	Triglycerides	CH3 CH2 twisting and bending	42, 43
1317	Histidine	CH_3_/CH_2_ twisting and wagging	39, 40
1339	Threonine	CH_3/_CH_2_ twisting and wagging	39, 40
1448	Lipids and proteins	CH_2_ and CH_2_CH_3_ bending, scissoring, and deformation	39, 40, 41, 42, 43
1516	Carotenoids	C–C stretching and C=C stretching	40, 41, 43
1551	Tryptophan	Symmetric breathing, C–C stretching	39, 40, 41
1576	DNA and NADH	Ring breathing modes	40, 41
1585	Phenylalanine	C–C bending	39, 40, 41
1604	Phenylalanine	Ring C–C stretching	39, 40, 41
1615	Tyrosine	C=C stretching	39, 40, 41
1657	Unsaturated lipids, PC, phosphatidylethanolamine	C=C stretching	40, 41, 42, 43
1670	Amide I	C=O stretching	41, 44

The Raman peak values for samples from both healthy and GDM were then used to compute Pearson correlation coefficients (Figure 1c) between each metabolite and the class (0 for healthy, 1 for GDM). For clarity and accuracy, redundant peaks were not used for the correlation, and only the most intense peak associated with each metabolite was used. Values between ± (0–0.29) suggest a weak correlation, ± (0.30–0.50) moderate correlation, and ± (0.51–1) suggest a strong correlation of variates to each other.^48^ Regardless of the sign, metabolites with high correlation coefficient values are more effective at differentiating between the healthy and GDM groups, suggesting a potential biomarker or metabolic signature associated with GDM. Conversely, negative correlations imply lower metabolite concentrations in GDM. We also investigated the correlations between first trimester metabolites and late second trimester blood glucose levels for the patients in the GDM and healthy cohorts (Figure 1d). While there was limited correlation for the healthy cohort, the GDM cohort had significant correlations between metabolites and late second trimester blood sugar levels. The most significant negative correlations (correlation value < −0.30) include correlations with lipids and fatty acids (1060, 1078, and 1173 cm^−1^), amide III (1242 cm^−1^), amino acids (1206 cm^−1^), and tyrosine (1615 cm^−1^). Significant positive correlations (correlation value >0.30) include correlations with membrane lipids (719 cm^−1^), cholesterol (701 cm^−1^), unsaturated lipids (1657 cm^−1^), and carotenoids (1155 and 1516 cm^−1^). The increased degree of correlation with the first trimester metabolites and blood sugar for GDM patients suggests that the degree of severity of GDM (as represented by the degree of hyperglycemia) can be influenced by metabolite factors early in pregnancy. Early intervention through diet, exercise, and treatment has been shown to reduce risk factors of GDM if begun early in pregnancy.^49^

**FIGURE 2:**
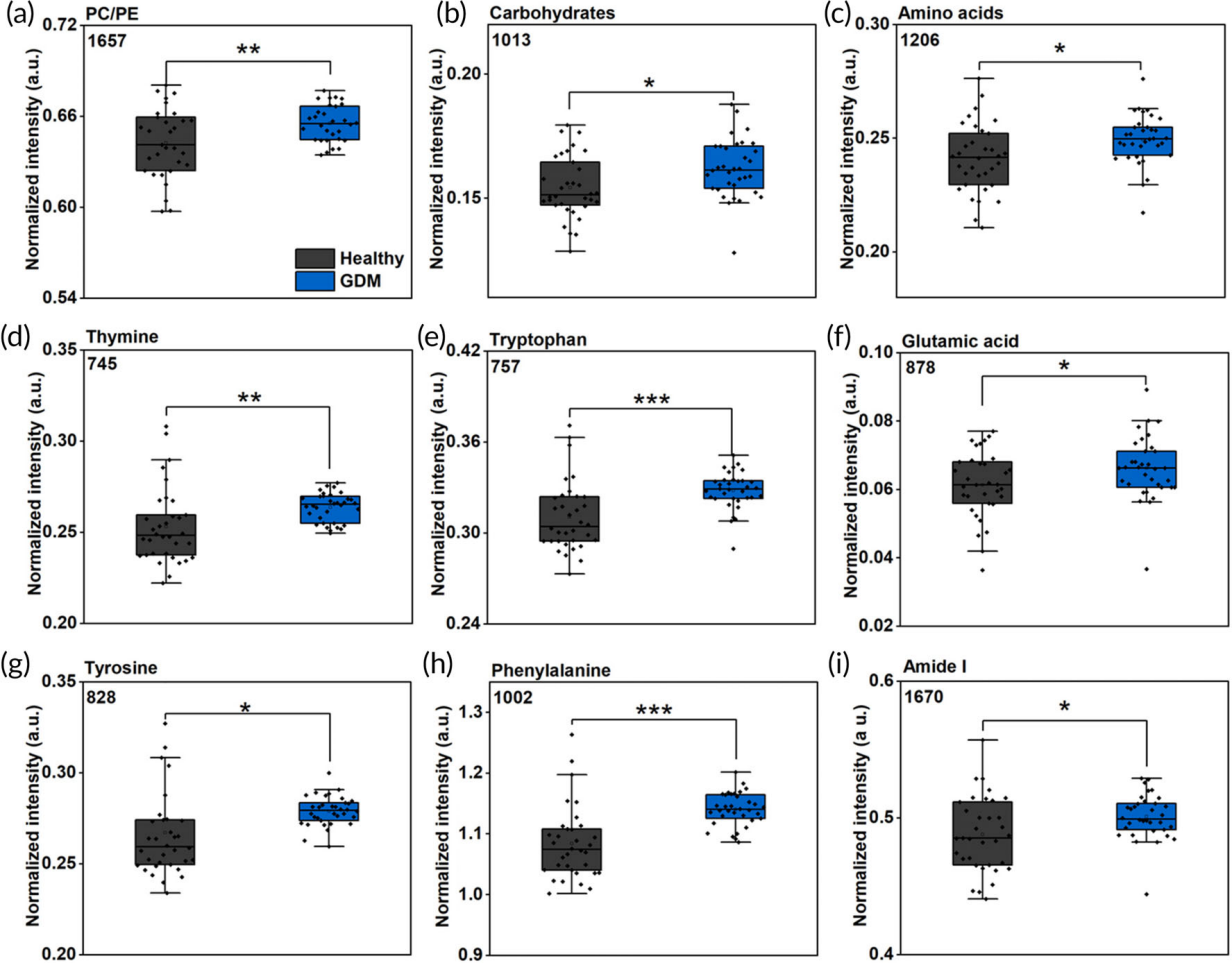
Key metabolites that distinguish gestational diabetes mellitus (GDM) (blue) from healthy (black) patients are presented in box plots (a–i). Metabolites increasing in GDM, including PC/PE (1657 cm^−1^), carbohydrates (1013 cm^−1^), amino acids (1206 cm^−1^), thymine (745 cm^−1^), tryptophan (757 cm^−1^), glutamic acid (878 cm^−1^), tyrosine (828 cm^−1^), phenylalanine (1002 cm^−1^), and amide I (1670 cm^−1^). Here, * represents a *p* value <0.05, ** represents a *p* value <0.01, and *** represents a *p* value <0.001. PC, phosphatidylcholine; PE, phosphatidylethanolamine.

Statistical analysis of our data using a two‐tailed student's *t*‐test identified nine statistically significant peaks (Figure 2) corresponding to different metabolites that support this assessment. Other peak distributions are included in Figure S3. We observed that the peaks corresponding to carbohydrates (1013 cm^−1^), unsaturated lipids such as phosphatidylcholine and phosphatidylethanolamine (PC/PE) (1657 cm^−1^), amino acids (AAs) (1206 cm^−1^) that also include thymine (745 cm^−1^), tryptophan (757 cm^−1^), glutamic acid (878 cm^−1^), tyrosine (828 cm^−1^), and phenylalanine (1002 cm^−1^), and protein‐related amide I (1670 cm^−1^) peak increased for the GDM cohort. Carbohydrates maintain the blood glucose level, and therefore an increase in the GDM cohort implies dysregulated carbohydrate metabolism, likely due to insulin resistance resulting in glucose intolerance.^50^ Alterations in lipid metabolism have also been reported in pregnancy, resulting in fatty acids (FAs) and phospholipids, among others, in maternal circulation, with a high likelihood of dyslipidemia in GDM patients.^51^ This may have been attributed to the increase in the 1657 cm^−1^ Raman peak in the GDM cohort. We also observe an increase in most AA peaks in the GDM cohort. AAs are necessary for fetal protein synthesis and are actively transported across the placenta to the fetus. An accumulation of AAs in maternal circulation suggests altered placental metabolism that limits nutrient transport across the placenta and has been reported in GDM.^52^ For example, tryptophan, an essential AA, is crucial for fetal growth and placental function. However, the tryptophan‐kynurenine catabolic pathway is altered in GDM,^53^ resulting in an elevation of xanthurenic acid, a metabolic intermediate that contributes to insulin resistance and may compromise the pancreatic β‐cell function.^54^ The inflammatory phenotype of GDM also contributes to the altered tryptophan‐kynurenine pathway.^55^ Our findings are aligned with literature evidence where high levels of tryptophan metabolites were observed in the urine of GDM patients throughout gestation,^55^ suggesting tryptophan may serve as a key early metabolic marker of GDM progression. An increase in glutamic acid, a non‐essential AA, observed in our study is likely attributable to the development of hyperglycemia and is associated with both GDM and type 1 and 2 diabetes.^56^, ^57^ An increase in glutamic acid may also result from altered glutamine‐glutamate exchange between the fetal liver and the placenta, which increases hepatic production of maternal glutamate.^58^ We also observed an increase in phenylalanine and tyrosine, AAs that are crucial during gestation primarily for protein synthesis. Indeed, patients with high phenylalanine and tyrosine in the first trimester later developed GDM, and these AAs have been associated with insulin resistance, obesity, and the future risk of diabetes.^59^ An increase in the amide I (1670 cm^−1^) protein peak in the GDM cohort likely correlates to the aberration in AA metabolism and dysregulated placental function leading to placental proteins entering maternal circulation. Further, insulin production abnormalities also lead to the subsequent increase in proteins associated with inflammation (such as C‐reactive proteins), glucose transport and regulation, and those involved in lipid metabolism (such as lipoproteins).^60^, ^61^ The higher level of DNA bases such as thymine in GDM may be correlated with the presence of cell‐free placental/fetal DNA in maternal circulation associated with GDM.^62^ Abnormalities in placental development in inflammatory condition can change placental cell composition, releasing fetal DNA from the placenta into maternal circulation.^37^

**FIGURE 3:**
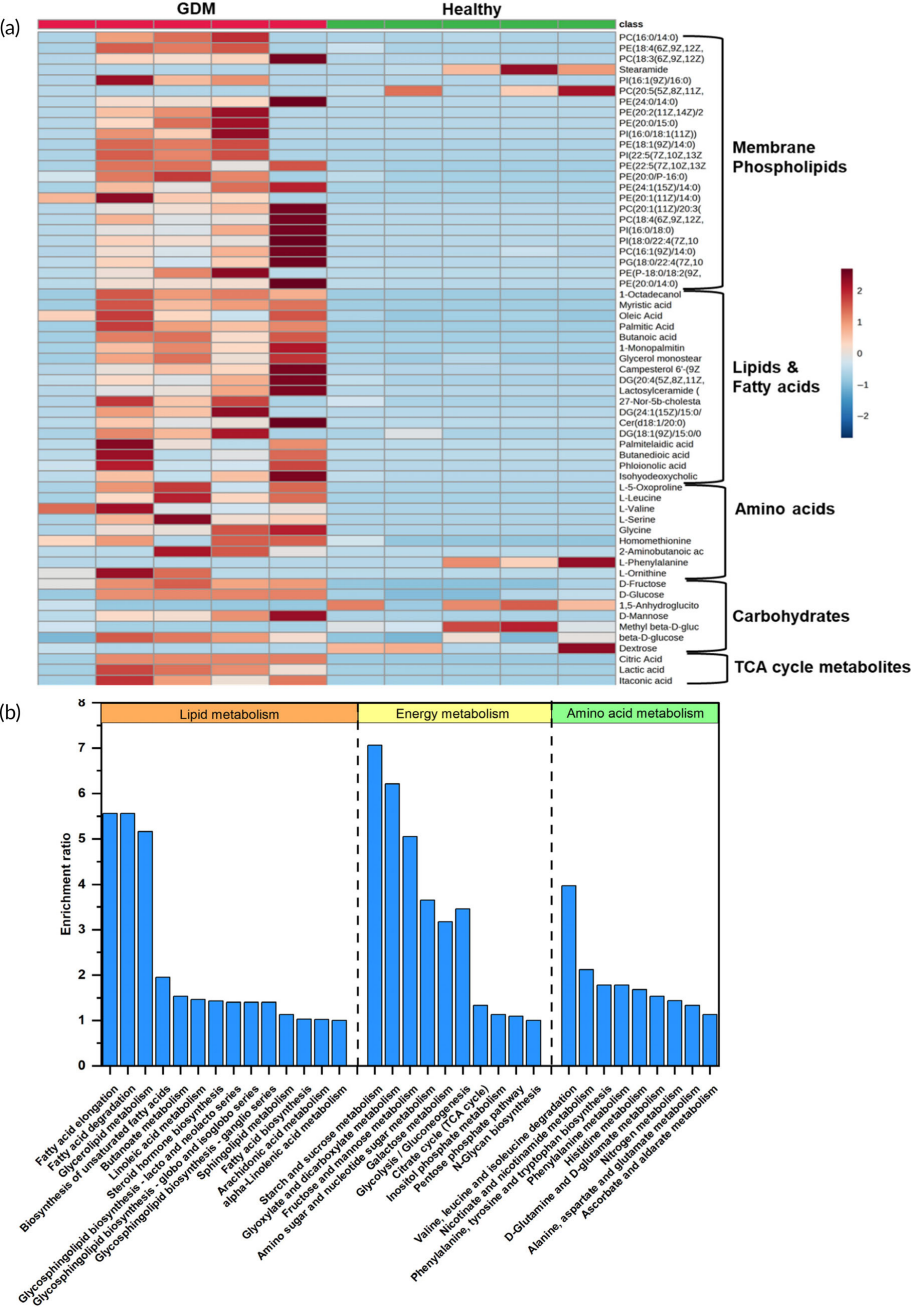
Mass spectrometry metabolomics identifies metabolites that distinguish gestational diabetes mellitus (GDM) from healthy. (a) Heatmap of statistically significant metabolites obtained from liquid chromatography–mass spectrometry (MS) and gas chromatography–MS of *n* = 5 patients per group. (b) Kyoto Encyclopedia of Genes and Genomes pathway enrichment analysis shows metabolic pathways that correspond to the metabolites enriched in the patient samples. PC, phosphatidylcholine; PE, phosphatidylethanolamine; TCA, tricarboxylic acid.

Our Raman findings were validated with MS metabolomics by performing gas chromatography–MS (GC–MS) and liquid chromatography–MS (LC–MS) on metabolites extracted from plasma. Compared to Raman analysis, larger sample volumes and labor‐intensive sample extraction are required for MS; however, MS approaches remain the gold standard in metabolite identification and have been leveraged for our study. The statistically significant metabolites observed in MS are represented as a heatmap in Figure 3a and are categorized into different classes for better visualization. Generally, most classes of metabolites demonstrate an increase in the GDM cohort relative to healthy, similar to the trends observed in Raman analysis. Most AAs increase in GDM similar to that observed in Raman analysis, except in L‐phenylalanine, which decreases. Some variations between trends observed in Raman and MS are expected, as MS identifies a much larger subset of AAs than Raman. Note that due to the high cost of MS measurements and the limited sample volumes available to us (MS requires 60 μL aliquots × multiple technical replicates), we measured a subset of the samples with MS with the sole purpose of validating Raman findings. Our previous studies also support that metabolic trends in MS correspond well with those measured with RS.^33^, ^36^

The metabolites identified from MS measurements enabled metabolic pathway analysis that are enriched in the pathophysiology of GDM. KEGG database from Metaboanalyst, a web‐based interface for analysis of metabolic data,^63^ identified 35 enriched pathways with an enrichment ratio above 1 (Figure 3b). The detailed list of MS metabolites associated with the respective pathways is shown in Table S3. The enriched pathways can be categorized into lipids, amino acids, and energy metabolism. Within lipid metabolism, pathways related to FAs, including FA elongation, degradation, and biosynthesis, were enriched. During the initiation of GDM, oxidative stress and mild inflammatory conditions lead to increased lipolysis and the formation of free FAs, resulting in insulin resistance and impaired glucose tolerance.^64^ Recent studies showed that lipotoxicity‐induced β cell dysfunction drives the development of GDM; the lipotoxicity arises from both the accumulation of lipid intermediates and endogenous lipids synthesized from excess glucose via lipogenesis.^65^ Further, the glycerolipid metabolic pathway is also enriched, which regulates the synthesis, breakdown, and storage of glycerolipids. The upregulation of this pathway suggests activated lipid storage that converges on the lipotoxicity discussed above. Linoleic and alpha‐linoleic acid metabolism are influenced by the endogenous FA pathway and are inversely associated with GDM progression.^66^ Pathways involving glycosphingolipids, which have a role in inflammation, are also enriched. The sphingolipid family can modulate the activity of receptor tyrosine kinases such as insulin receptor and is involved in the development of insulin resistance.^67^ Other enriched FAs include arachidonic acids that are essential for fetal development and have been associated with GDM progression.^68^

Within energy metabolism, starch and sucrose metabolism showed the highest enrichment ratio. Starch and sucrose are common dietary carbohydrates that are metabolized to glucose, contributing to the pathogenesis of GDM. While this pathway is relevant in both normal and GDM pregnancies, the consequences of carbohydrate intake can be more significant due to insulin resistance. Other pathways include those involving glyoxylate, oxalate, and dicarboxylates. Glyoxylates can be used for the synthesis of two‐carbon compounds (gluconeogenesis) and dicarboxylates such as malate and fumarate can enter the tricarboxylic acid (TCA) cycle to generate energy. In GDM, high glucose concentration interferes with metabolic changes involving insulin resistance, directly impacting glyoxylate and dicarboxylate metabolism.^69^ Sugars such as fructose and mannose and their intermediates enter glycolysis, and while fructose does not directly impact GDM, excessive dietary intake contributes to glucose dysregulation. Further, fructose‐6‐phosphate, which is an intermediate of mannose, can enter the hexosamine biosynthesis pathway (HBP) associated with insulin resistance.^70^ GDM showed an enrichment of amino sugar and nucleotide sugar metabolism. Amino sugars such as glucosamine and galactosamine are synthesized in the body and are involved in the production of glycoproteins, glycolipids, and proteoglycans that contribute to insulin resistance via HBP. Galactose metabolism involves the conversion of galactose into glucose. High levels of galactose, the most abundant sugar in the human diet, lead to insulin resistance and the subsequent development of diabetes.^71^ Associated with galactose metabolism, we also find glycolysis/gluconeogenesis is elevated in GDM. Glycolysis breaks down glucose, and gluconeogenesis involves the synthesis of glucose from non‐carbohydrate precursors. Insulin resistance triggers higher glycolytic and gluconeogenic activity as cells attempt to meet their energy needs.^72^ Other energy metabolism pathways that are essential in GDM include the TCA cycle, which is central to energy production from carbohydrates, fats, and amino acids. Since the TCA cycle is interconnected with other metabolic pathways such as glycolysis, fatty acid synthesis, etc., changes in these pathways directly impact the TCA cycle. Further, a reduced TCA cycle flux is correlated with the defect in mitochondrial dysfunction that is observed in type 2 diabetes.^73^ The pentose phosphate pathway (PPP, phosphogluconate pathway), which runs parallel to glycolysis, is a major metabolic driver in the synthesis of coenzymes, nucleotides, and the production of nicotinamide adenine dinucleotide phosphate(NADP). PPP acts as a “protective” mechanism against hyperglycemia‐induced damage and limits the processing of glycolytic intermediates into harmful metabolic products. Downregulation of genes associated with PPP in the placental tissue of GDM patients indicates that elevated maternal glucose levels decrease the protective activity of PPP by increasing the methylation of specific genes.^74^ N‐glycan biosynthesis is a complex and highly regulated process in which glycans, which are complex sugars composed of glucose, mannose, and N‐acetylglucosamine units, are added to proteins. Alterations in N‐glycan biosynthesis are directly associated with hyperglycemia, insulin resistance, and the onset of diabetes.^75^

Among pathways associated with AA metabolism, valine, leucine, and isoleucine degradation, which are branched‐chain amino acids (BCAAs), were highly enriched. BCAAs induce insulin resistance in different metabolic tissues.^76^ For example, the accumulation of BCAAs in skeletal muscles promotes fatty acid uptake, and induces incomplete oxidation of fatty acids, resulting in insulin resistance. The decomposition of branched‐chain α‐keto acids in the liver impairs the TCA cycle, giving rise to the accumulation of incomplete oxidation products, mitochondrial stress, and insulin resistance. BCAAs also enable glucose uptake by the liver and increase glycogen synthesis in an insulin‐independent manner, highlighting the many roles of BCAAs in GDM pathogenesis. Affected pathways in GDM also include nicotinate and nicotinamide, which are precursors for the coenzymes nicotinamide adenine dinucleotide (NAD) and NADP. These metabolites are intertwined in cellular respiration, glycolysis, and the TCA cycle that drives glucose metabolism.^77^ Other altered AA pathways that play a major role include phenylalanine, tyrosine, and tryptophan biosynthesis, as well as those involving histidine, alanine, aspartate, and glutamate. These metabolites are essential for fetal growth and their dysregulation is strongly associated with GDM, as explained above with our Raman analysis in Figure 2.

**FIGURE 4:**
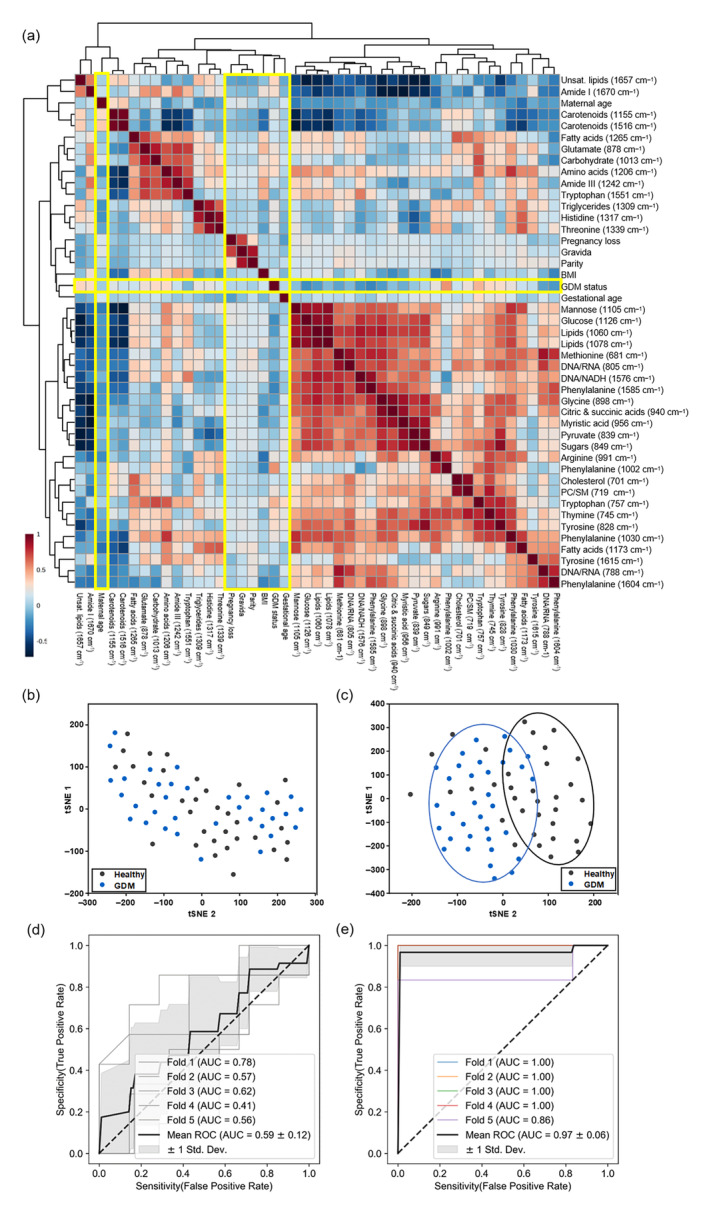
(a) Pearson correlation heatmap between Raman spectroscopy (RS) peak values and normalized clinical data for (*n* = 34) healthy and (*n* = 34) gestational diabetes mellitus (GDM) patients. Clinical factors and the GDM status are highlighted. (b) Principal component analysis (PCA)‐t‐stochastic neighbor embedding (tSNE) plot using clinical data to cluster healthy and GDM patient samples. (c) PCA‐tSNE plot using RS data to cluster healthy and patient GDM samples. (d) Area under the curve (AUC)‐reciever operating characteristic (ROC) curves for support vector machine (SVM) (radial basis function kernel) binary classification analysis using clinical data with five‐fold cross validation. (e) AUC‐ROC curves for SVM (second degree polynomial kernel) binary classification analysis using RS data with five‐fold cross validation.

These metabolic insights in the first trimester indicate that GDM is a highly metabolically active disorder. Therefore, we conceded that predictive models can be developed based on our RS metabolic data to predict the onset of GDM. We compared our approach to the use of patient clinical information, that is, demographic and obstetric data alone. Pearson's correlation analysis was performed on Raman peaks and their associated metabolites with patient clinical data and the GDM status (Figure 4a). The numeric correlation values of this heatmap are provided in Figure S4. Values between ± (0–0.29) suggest a weak correlation, ± (0.30–0.50) moderate correlation, and ± (0.51–1) suggest a strong correlation of variates to each other. Patient demographic data (BMI and maternal age) had minimal correlation to GDM status, which is consistent with the high *p* value of these demographics noted in Table 1. The patient obstetric data (gravida, parity, gestational age, and pregnancy loss) also had minimal correlation with the GDM status or any Raman peak. Whereas prior studies with large patient cohorts suggest that a higher gravida is a risk factor for GDM,^78^ the lack of correlation in our study is likely due to the limited sample size.

Moderate to strong correlations were observed between RS metabolites and both demographic and obstetric factors. For example, BMI was negatively correlated to carotenoid‐associated peaks (1155 and 1516 cm^−1^), and arginine (991 cm^−1^), and positively correlated to glutamate (878 cm^−1^) and the amide III band (1242 cm^−1^). However, as noted by the limited cross‐class significance and weak Pearson correlations, patient demographics are not sufficient to separate the classes. We employed principal component analysis (PCA) and t‐distributed stochastic neighbor embedding (tSNE) together (PCA‐tSNE) to visualize the unsupervised differentiation between the patients. 2 principal components (PCs) were used for the clinical data, and 10 PCs were used for the RS data. When clinical data that include demographic and obstetric data were used (Figure 4b), no differentiation was observed between the two cohorts. However, due to the stronger correlations and statistical significance between the Raman metabolic data and the GDM status, the RS peak values clearly differentiated the healthy and GDM cohorts (Figure 4c). PCA scatter plot for clinical data, RS metabolites, and PCA loading plots are included in Figure S5. We then used the Raman and clinical data to build supervised classification models to determine the accuracy of the separation observed in PCA‐tSNE. Given the smaller sample size in this study, we limited overfitting by reducing the feature count by removing redundant peaks and only using the most intense peak associated with each metabolite. This approach reduced the feature number from 39 peaks to 30 (Table S4). We then trained SVM models with a linear, polynomial, and radial basis function (RBF) kernels, optimizing model hyperparameters with an exhaustive grid search. This was done in Scikit‐Learn with both five‐fold cross‐validation (CV) and leave‐one‐out CV (LOOCV). The performance and hyperparameters for all models are listed in Table S5. Even with optimization, the best‐performing clinical data model (RBF kernel) was not able to make accurate predictions, with an average five‐fold CV AUC of 0.59 ± 0.12 (Figure 4d), an LOOCV AUC of 0.60, a five‐fold CV accuracy of 61.7% ± 9.6%, and a LOOCV accuracy of 60.3% ± 48.9%, indistinguishable from a random guess. However, the best‐performing Raman peak model (second degree polynomial kernel) had a five‐fold CV AUC of 0.97 ± 0.06 (Figure 4e), a five‐fold CV accuracy of 95.5% ± 3.7%, a LOOCV AUC of 0.96 (Figure S6a), and a LOOCV accuracy of 95.6% ± 20.5%. We inspected the performance of the SVM models to understand how they were leveraging the data set. We investigated the linear coefficients of the linear kernel version of the SVM model, which had a reduced performance compared to the polynomial kernel model (Figure S7a,b) but can be more easily inspected in linear space. The linear coefficients for the five‐fold CV (Figure S7c) and LOOCV (Figure S7d) match the correlations found in Figure 1c, with positive coefficients associating the feature with GDM patient samples and negative coefficients associated with healthy patient samples. The top‐three Raman features that were associated with GDM were the 1002, 1206, and 1657 cm^−1^ peaks, while the top‐three features associated with healthy patients were the 898, 1126, and 1265 cm^−1^ peaks. We also investigated permutation importance for the five‐fold CV, which measures the impact on model accuracy from perturbing selected features before making predictions. This method has the advantage of being model agnostic, allowing for inspection of both linear and nonlinear kernels. The permutation importance for the linear model (Figure S7e) suggested that 11 out of the 30 selected features had a negligible importance with their standard deviation crossing the *y* axis. This suggests that either these features can be neglected, or that some of these features are overly correlated. For inspection of the polynomial model permutation importance (Figure S6b), we found that 21 out of 30 peaks were effectively unimportant, despite the increase in accuracy. This does suggest that the observed improvements in CV accuracy from the linear kernel (which has a five‐fold CV of 92.6% ± 4.5%) may be at the cost of generalization. Our data suggest that Raman metabolic profiling offers the benefit of accurate risk stratification of GDM even with a small sample size compared to population‐wide studies that require large patient cohorts to predict GDM risk. Our RS‐based approach also offers higher accuracy than single biomarker models in predicting the risk of GDM in the first trimester.^79^ The limited improvement from model optimization does suggest that larger data sets will be necessary to improve model performance.

**FIGURE 5:**
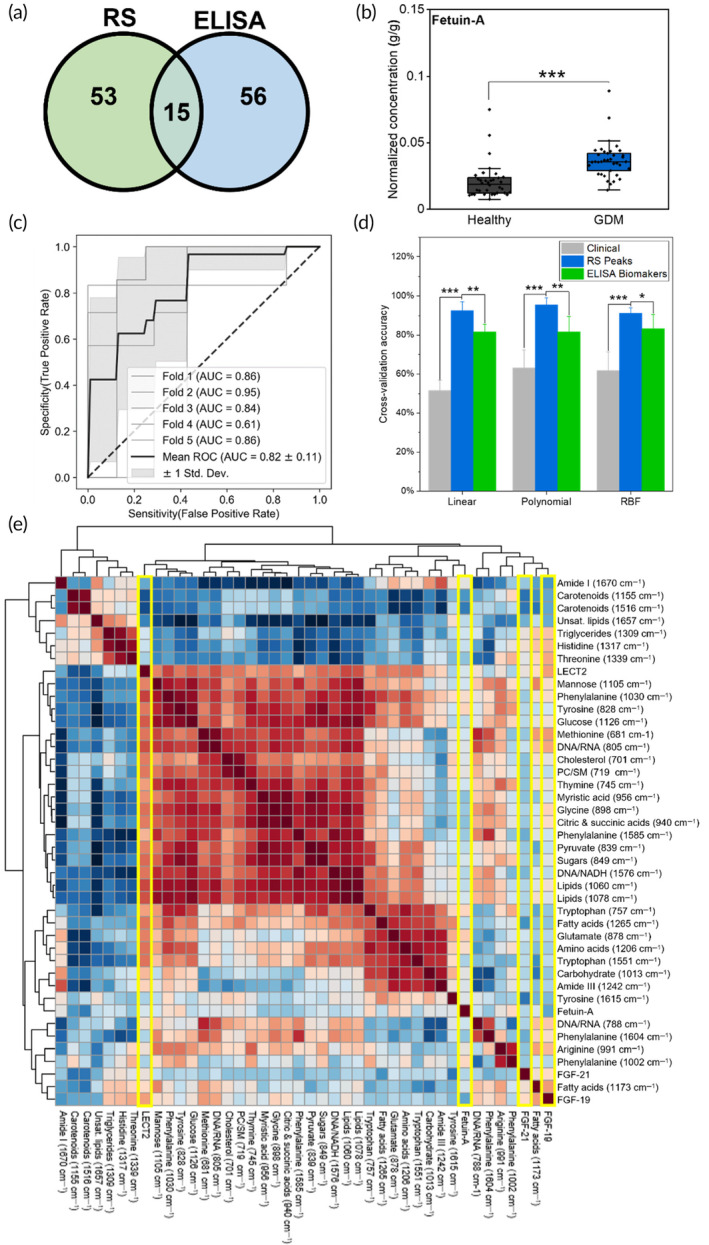
(a) Quantity of plasma samples for Raman spectroscopy (RS) and enzyme‐linked immunosorbent assay (ELISA) protein biomarker analysis. Fifty‐six patient samples had sufficient volume for ELISA analysis, 53 patient samples had sufficient volume for RS analysis, and 15 samples had sufficient volume to perform both analyses. (b) Protein expression for fetuin‐A for *n* = 37 gestational diabetes mellitus (GDM) and *n* = 34 healthy patients normalized to total protein content. (c) Area under the curve (AUC)‐reciever operator curve (ROC) for support vector machine (SVM) (radial basis function kernel) binary classification analysis trained on protein markers for *n* = 37 GDM and *n* = 34 healthy patients. (d) Five‐fold cross‐validation accuracies for SVM binary classification models trained on clinical, RS peak, and ELISA biomarker data sets using the linear, polynomial, and radial basis function kernels. Error bars indicate 1 SD. (e) Pearson correlation heatmap between RS peak values and normalized ELISA data of all four proteins (fetuin‐A, leukocyte cell‐derived chemotaxin 2 [LECT2], fibroblast growth factor 19 [FGF‐19], and fibroblast growth factor 21 [FGF‐21]) for *n* = 8 healthy and *n* = 7 GDM patients. Proteins are highlighted. Here, * represents a *p* value <0.05, ** represents a *p* value <0.01, and *** represents a *p* value <0.001.

We further conceded that identifying protein biomarkers that complement Raman metabolic data could enable a powerful integrated platform for early GDM screening. Four candidate proteins that are correlated to GDM, including fetuin‐A, leukocyte cell‐derived chemotaxin 2 (LECT2), fibroblast growth factor 19 (FGF‐19), and fibroblast growth factor 21 (FGF‐21) were assessed with ELISA in a subset of the patient samples. Protein concentrations were normalized to the total sample protein with bicinchoninic acid (BCA) assay. Among the proteins assessed, fetuin‐A blocks insulin receptors and has shown strong correlations to GDM onset.^80^ LECT2 is a hepatokine linked to insulin resistance and is correlated with type 2 diabetic patients.^81^ FGF‐19 is negatively correlated with GDM,^82^ while FGF‐21 is positively correlated with insulin resistance and type 2 diabetes.^82^ Protein analysis of fetuin‐A in *n* = 37 GDM patients and *n* = 34 healthy control patients (Figure 5a) had significant difference, *p* value <0.001, between the two cohorts (Figure 5b); the other proteins had reduced or no significant differences (Figure S8). Using all four proteins as a panel to train and optimize SVM models, we found that the RBF kernel was optimal. We achieved a five‐fold CV AUC of 0.82 ± 0.11 (Figure 5c), a five‐fold CV accuracy of 83.1% ± 7.3%, a LOOCV AUC of 0.86 (Figure S9a), and an LOOCV of 85.9% ± 34.8%. Investigating the permutation importance of the RBF model, we found that only fetuin‐A had a robust predictive impact (Figure S9b). The linear version of the model trained on the same data set (Figure S10) also indicates that the other biomarkers had negligible importance for training the predictive models. Comparing the clinical, RS peak, and ELISA data sets across SVM models (Figure 5d), we found that both the RS and ELISA models were significantly more accurate than the clinical model, and that the RS model was more accurate than the ELISA model across kernels (Figure S9c). While the increased variance of the LOOCV reduced this significance in that validation scheme (Figure S9b), for both the five‐fold CV and LOOCV the best‐performing RS model (second degree polynomial) was more accurate than the best‐performing ELISA model (RBF), with a *p* value <0.01 for the five‐fold CV, and a *p* value <0.05 for the LOOCV. This comparison serves to both validate the potential predictive ability of the RS metabolic profile, and to show that a combination of proteins and metabolites via ML analysis can be leveraged to generate risk scores for GDM assessment. We further compared a subset of samples (*n* = 8 healthy and *n* = 7 GDM) that underwent both Raman imaging and ELISA of fetuin‐A. PCA clustering for the two different data sets (Figure S11a,b) showed slightly better separation of cohorts based on fetuin‐A analysis relative to Raman metabolites. A Pearson correlation heatmap for this subset of samples (*n* = 8 healthy and *n* = 7 GDM) correlating Raman metabolites to all four proteins (fetuin‐A, LECT2, FGF‐19, and FGF‐21) is shown in Figure 5e. The numeric correlation values are represented in Figure S12 and a feature heatmap for the sample subset is shown in Figure S13. Interestingly, we found that fetuin‐A had a moderate negative correlation to carotenoid peaks (1516 cm^−1^) but had a limited correlation with the metabolite data overall. A previous robust study showed a similar independence from metabolic factors for fetuin‐A.^83^ This orthogonality from metabolic factors suggests that fetuin‐A would be an ideal candidate for protein screening to complement a metabolic panel. FGF‐21 also showed moderate negative correlations with carotenoids. LECT2 had the strongest correlations to metabolites, including strong positive correlations with fatty acid and lipid peaks (1060 and 1078 cm^−1^), and moderate positive correlations with tryptophan (757 and 1551 cm^−1^), carbohydrate and sugar peaks (849, 1013, 1105, and 1126 cm^−1^) and some other peaks. LECT2 also had a negative correlation to carotenoids. Previous literature findings have shown negative correlation of LECT2 with high‐density lipoprotein‐cholesterol levels in type 2 diabetes patients,^81^, ^84^ but a positive correlation with low‐density lipoprotein‐cholesterol. However, the lack of correlation between BMI and the Raman peaks correlated to LECT2 in the broader cohort shown in Figure 4a and no significant difference in LECT2 between the healthy and GDM cohorts (Figure S8), indicates our results do not support LECT2 as a potential marker for GDM assessment. FGF‐19 showed correlations to many RS metabolites, including positive correlations to multiple amino acid peaks (681, 991, 1317, and 1339 cm^−1^) and DNA‐related bands (788 and 805 cm^−1^). FGF‐19 is linked to hepatic protein synthesis, which may explain the relationship between the increased amino acids and FGF‐19 expression.^85^ Further, a statistically significant difference was observed for FGF‐19 between the healthy and GDM cohorts (Figure S8) suggesting FGF‐19 analysis in conjunction with fetuin‐A and a Raman‐based metabolic panel may have high predictive value early in pregnancy.

## 3 CONCLUSIONS

In summary, this study demonstrates the ability of RS as a rapid and low‐cost tool to measure the circulating metabolite profile in pregnant patient plasma in the first trimester, enabling early prediction of patients at risk of developing GDM. Our quantitative analysis shows several metabolites, including phospholipids, carbohydrates, and amino acids, differentiated healthy from GDM patients early in pregnancy. MS metabolomics validated Raman findings and identified key metabolic pathways that are enriched in GDM, including fatty acid degradation and elongation, glycolysis and metabolism of other sugars, and branched‐chain amino acid metabolism. Combining Raman metabolites in the first trimester with classical ML models (PCA‐tSNE and SVM) allowed the differentiation of healthy and GDM patient cohorts with a five‐fold CV AUC of 0.97 superseding current predictive models based on single protein markers or metadata analysis with patient clinical and demographic data only. Further, analysis of a panel of proteins identified fetuin‐A with a strong significance to GDM in the first trimester and a five‐fold CV AUC of 0.82. A correlation analysis of proteins to RS metabolites indicated that fetuin‐A, LECT2, and FGF‐19 all had moderate to strong correlations to metabolites. Our data suggest that a combined metabolic and protein analysis with simple ML models would enable a powerful predictive tool for risk stratification of GDM early in pregnancy, complementing current prenatal care and blood sugar tests offered later in pregnancy. Further, our study can also be translated to rural settings since RS is low‐cost, label‐free, and extraction‐free, allowing affordable GDM screening. Future studies with larger cohorts of patients and a larger array of proteins combined with sophisticated ML models could improve the accuracy of our approach and enable predictive risk scoring that surpasses current clinical measures for GDM risk analyses.

## 4 EXPERIMENTAL METHODS

### 4.1 Inclusion or exclusion criteria for collection of patient samples and ethical statement

All patients provided informed consent to have blood collected by the PFTB from the UI Hospitals & Clinics Department of Obstetrics and Gynecology. This research was reviewed and approved by the Institutional Review Board (IRB#200910784). Blood was collected in anticoagulant citrate dextrose A (ACD‐A) tubes (Becton Dickinson), centrifuged, and plasma was aliquoted, snap‐frozen, and stored at −80°C. The plasma samples from the patients and associated clinical data were provided to us coded with all of the Health Insurance Portability and Accountability Act (HIPAA) identifiers removed.^86^ The PFTB at UI, which is an academic biobank, prospectively collects maternal biofluids (blood and urine) throughout gestation as well as at delivery (amniotic fluid and placenta). PFTB continuously recruits participants serving as a cross‐sectional clinical datamart that provides samples for research and educational purposes. Therefore, patients were not specifically recruited for this study. All subjects used in this study were pregnant females between the ages of 18 and 50 with singleton gestation. All procedures followed were in accordance with the ethical standards of the IRB committee on human experimentation and aligned with the Helsinki guidelines of 1975.

### 4.2 Sample preparation for Raman mapping and analysis

The plasma samples were thawed, and 3 μL was aliquoted on 2 mm by 2 mm calcium fluoride disks and dried in a 37°C oven for 20 min before measurement. The RS imaging of the dried plasma was conducted using a Renishaw inVia Raman confocal microscope equipped with a 785 nm laser source with WiRE 5.4 software. The laser power applied to the sample was 40 mW at the point of excitation using a Lexica 50× objective. The spectrophotometer was calibrated daily using a standard silicon wafer at a wavelength of 520.5 nm. Static scans were taken with a spectral window ranging from 600 to 1700 cm^−1^ with 5 s of exposure time and two accumulations. Line maps were recorded for each sample with a step size of 1 μm, and 100 spectra were obtained from 10 line maps per sample. Line maps were conducted on smooth stretches of the dried plasma film, ensuring that the entirety of the line map was in focus. The 10 line maps were taken in spatially distinct areas of the film for comprehensive sampling. In addition, we avoided areas prone to artifacts or surface irregularities that could affect spectral quality. A custom MATLAB (R2021b) code was used to process the raw spectra, including cosmic ray removal, baseline correction, smoothing, and SNV method normalization. The spectra were smoothed using a Savitzky and Golay filter with a ninth‐order polynomial and coefficient value of 27 points, and the fluorescent background was removed using an 11th‐order modified polynomial fit with a threshold value of 0.0001. t‐distributed stochastic neighbor embedding (tSNE), a non‐linear and unsupervised multivariate dimensionality reduction method, was used to show group separation using the tSNE function of the MATLAB Statistics and Machine Learning Toolbox. PCA was used to reduce the spectra dimensionality prior to tSNE fitting (e.g., PCA‐tSNE). The parameters included the “exact” algorithm with a Chebychev distance function, a perplexity value of 30 and an exaggeration value of 3. The PCA components varied by figure and are included in the text.

### 4.3 Mass spectrometry metabolomics

Plasma sample preparation was performed following a modified methanolic extraction method established by Jiye et al.^87^ The detailed procedure was previously reported by our group.^36^ Briefly, 50 μL of each sample were spiked with internal standards (nonadecanoic acid and ribitol), and the extraction was initiated with the addition of an ice‐cold LC–MS‐grade methanol–water mixture (8:2). Samples were kept on ice with intermittent vortexing followed by sonication (ice‐cold water bath) and vortexed again before centrifuging to finally collect the supernatant. The pellets were re‐extracted with ice‐cold methanol, and the supernatants were pooled together. The pooled supernatant extracts were then filtered with 0.2 μm centrifugal filters. The samples were submitted to the W.M. Keck Metabolomics Research Laboratory (Office of Biotechnology, Iowa State University, Ames, IA) for nontargeted metabolomic analysis (GC–MS/LC–MS). Metabolite peaks were identified using accurate mass spectral analysis compared to the METLIN database.

### 4.4 Enzyme‐linked immunosorbent assay

ELISA assay was performed using maternal plasma following the manufacturer's protocol. The following ELISAs were performed: alpha‐2HS‐Gycoprotein (sample diluted 1:50,000, catalog# EHAHSG), LECT2 (sample diluted 1:20, BioVendor catalog # RD191370200R), FGF‐19 (non‐diluted sample, R & D Systems catalog# DF1900), and FGF‐21 (sample diluted 1:50, R&D Systems catalog# DF2100). Absorbance was recorded using a BioRad xMark Spectrophotometer. Total protein was quantified using a BCA assay (sample diluted 1:50, Pierce Catalog# 23225).

### 4.5 MetaboAnalyst

MetaboAnalyst 5.0, a web‐based platform, was used for the data analysis, correlation analysis, enrichment analysis, and correlation heatmaps. The heatmaps and correlation analysis for the Raman and MS data were created using the Statistical analysis (one factor) module. The correlation graphs were prepared using Statistical analysis (metadata table). Statistical analysis (one factor) module with default MetaboAnalyst parameters such as Pearson *r* distance measure were used to generate heatmaps. The detailed metabolic pathways for MS data were created using the Enrichment Analysis module with KEGG pathway, ID type as compound names, and feature type as metabolites.

### 4.6 SVM model training

The Scikit‐learn (sklearn) 1.2.0 package for Python 3.10.6 was used for supervised ML model training and model analysis.^88^ The sklearn svm.SVC function was used for SVM binary classification model training. For all model regressions, a gamma value of “scale” and a decision shape of “one‐vs‐one” were used. Regressions were conducted for the linear, polynomial, and RBF kernels, with the optimal regularization parameter (*c*‐value) and polynomial degree (for using the polynomial kernel) determined through exhaustive grid search for both five‐fold and leave‐one‐out (LOO) CV methods. A class weight of “balanced” was used to account for any uneven distributions between classes. All other function parameters were left as default.

### 4.7 SVM model evaluation

To evaluate the performance of the SVM model on the data set, both five‐fold and LOO CV methods were used. For five‐fold CV, the sklearn.model_selection.KFold function was used to split the data set into five non‐overlapping train/test sets. For LOO CV, sklearn.model_selection.LeaveOneOut was used to split the data set into a number of train/test sets equal to the number of samples, with a single sample as the test sample for each fold. Using these train/test sets, both model CV accuracy and AUC‐ROC curves were evaluated to determine model performance. To understand how the SVM model was utilizing the data set features, the model coefficients from the linear coefficients were averaged for each fold and reported. In addition, permutation feature importance analysis was used for both linear and polynomial SVM models, using the five‐fold CV train/test sets and the sklearn.inspection.permutation_importance function. The permutation_importance function was used to evaluate each fold with 30 repeats, and the mean decrease in accuracy from the permutation of each fold was reported.

### 4.8 Statistical analysis

The data are represented as the mean ± standard error. Unpaired, two‐sided, homoscedastic Student's *t*‐tests were used in excel to generate *p* value. *p* Values of <0.05, <0.01, and <0.001 are indicated as *, **, and ***.

## AUTHOR CONTRIBUTIONS

**Ansuja P. Mathew:** Formal analysis; methodology; writing – original draft; writing – review and editing. **Gabriel Cutshaw:** Formal analysis; methodology; writing – original draft; writing – review and editing. **Olivia Appel:** Data curation; formal analysis. **Meghan Funk:** Data curation; investigation. **Lilly Synan:** Data curation; formal analysis. **Joshua Waite:** Software; validation. **Saman Ghazvini:** Formal analysis; investigation. **Xiaona Wen:** Formal analysis; supervision. **Soumik Sarkar:** Software; validation. **Mark Santillan:** Resources; supervision; writing – review and editing. **Donna Santillan:** Resources; supervision; writing – review and editing. **Rizia Bardhan:** Conceptualization; resources; supervision; writing – review and editing.

## CONFLICT OF INTEREST STATEMENT

The authors declare no conflict of interest.

## Supporting information


**Data S1.** Clinical information tables for individual healthy and GDM patients; Raman spectra of individual healthy and GDM patients; Boxplots of additional Raman metabolites; Raman metabolites associated with KEGG metabolic pathway; Pearson correlation heatmap between RS and clinical data; Feature list for SVM training; Performance of SVM models; Cross‐validation of SVM model; Protein analysis with ELISA; PCA analysis for RS and protein subset; Pearson correlation heatmap between RS and proteins; Heatmap for RS peak and protein correlation analysis. This material is available free of charge via the Internet https://www.aiche.org/publications/journals/bioengineering-translational-medicine.

## Data Availability

The data supporting this study's findings are available from the corresponding author upon reasonable request.
