# Supplementary material for: Diagnosis of pregnancy disorder in the first‐trimester patient plasma with Raman spectroscopy and protein analysis
Source: Bioeng Transl Med. 2024 Jul 16;9(6):e10691. doi: 10.1002/btm2.10691 (PMC11558203; doi:10.1002/btm2.10691)
Supplement: Supplementary file 1 — Data S1. Clinical information tables for individual healthy and GDM patients; Raman spectra of individual healthy and GDM patients; Boxplots of additional Raman metabolites; Raman metabolites associated with KEGG metabolic pathway; Pearson correlation heatmap between RS and clinical data; Feature list for SVM training; Performance of SVM models; Cross‐validation of SVM model; Protein analysis with ELISA; PCA analysis for RS and protein subset; Pearson correlation heatmap between RS and proteins; Heatmap for RS peak and protein correlation analysis. This material is available free of charge via the Internet https://www.aiche.org/publications/journals/bioengineering-translational-medicine. [file BTM2-9-e10691-s001.docx]

**Supporting Information**

**Diagnosis of pregnancy disorder in the first-trimester patient plasma with Raman spectroscopy and protein analysis**

Ansuja P. Mathew, ^†,1,2^ Gabriel Cutshaw, ^†,1,2^ Olivia Appel,^1,2^ Lilly Synan,^1,2^ JoshuaWaite,^3^ Saman Ghazvini,^1,2^ Xiaona Wen,^2^ Soumik Sarkar,^3^ Meghan Funk,^4^ Mark Santillan,^4^ Donna Santillan,^4^ Rizia Bardhan*^1,2^

^1^Department of Chemical and Biological Engineering, Iowa State University, Ames, Iowa, USA ^2^Nanovaccine Institute, Iowa State University, Ames, Iowa, USA

^3^Department of Mechanical Engineering, Iowa State University, Ames, Iowa, USA

^4^Department of Obstetrics and Gynecology, Carver College of Medicine, University of Iowa Hospitals & Clinics, Iowa City, Iowa, USA

† These authors have contributed equally

* Corresponding author: [rbardhan@iastate.edu](mailto:rbardhan@iastate.edu)

**Table of Contents**

**Figure S1**. Representative images of the samples and corresponding sample PCA from healthy and GDM patients…………………………………………………………………………………3

**Table S1**. Healthy patients’ clinical information…………………………………………………..4

**Table S2**. GDM patient's clinical information……………………………………………………..6

**Figure S2**. Raman spectra of all healthy and GDM patient samples..……………………………...7

**Figure S3**. Representation of Raman metabolites as box plots that distinguish GDM **(blue)** from healthy **(black)** patients..………………………………………………...………………………...8

**Table S3**. List of key metabolites from MS KEGG pathway enrichment analysis. ..……………..10

**Figure S4.** Pearson correlation values for Raman and clinical data. ..…………………………...12

**Figure S5.** PCA scatter plots for clinical data, RS data, and PCA Loading Plots………………13

**Table S4.** Truncated feature list used for SVM classification……………………………………14

**Table S5.** Summary of SVM model hyperparameter optimization and performance……………15

**Figure S6.** Performance of optimized polynomial kernel SVM model trained on RS peak data to differentiate (n=34) GDM and (n=34) healthy samples………………………………………….15

**Figure S7.** Performance of optimized linear kernel SVM model trained on RS peak data to differentiate (n=34) GDM and (n=34) healthy samples……………………….…………………16

**Figure S8.** Protein expression from ELISA for GDM and healthy patients……………………....17

**Figure S9.** (a-b) Performance of optimized radial basis function kernel SVM model trained on ELISA biomarker data to differentiate (n=37) GDM and (n=34) healthy samples) and leave-one-out cross-validation accuracies for SVM binary classification models trained on clinical, RS peak and ELISA biomarker datasets…………………………………………………….……………..17

**Figure S10.** Performance of optimized linear kernel SVM model trained on ELISA biomarker data to differentiate (n=37) GDM and (n=34) healthy samples………………………………………..18

**Figure S11.** PCA analysis plot comparing PC1 and PC2 using ELISA data and RS data………19

**Figure S12.** Numeric Pearson correlation heatmap between RS peak values (unit = cm^-1^) and normalized protein biomarker data……………………………………………………………….20

**Figure S13.** Feature intensity heatmap for the (n=7) GDM and (n=8) healthy patient samples used for RS peak and biomarker correlation analysis………………………………………………….21


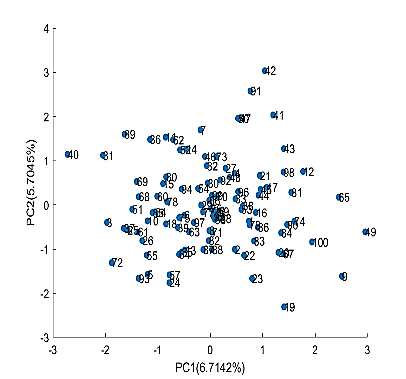

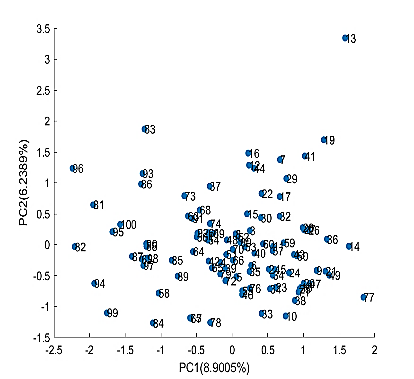

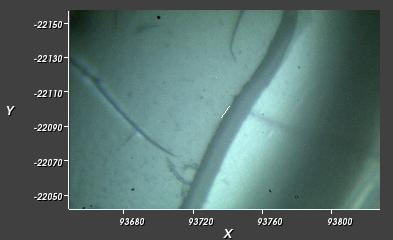

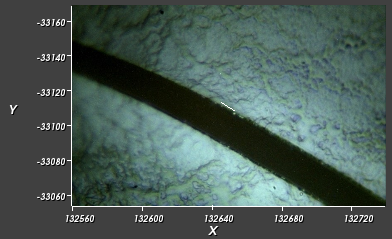

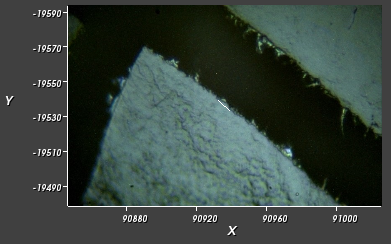

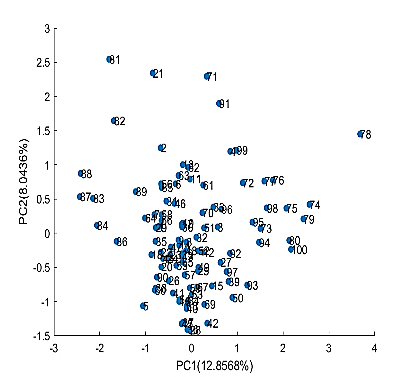

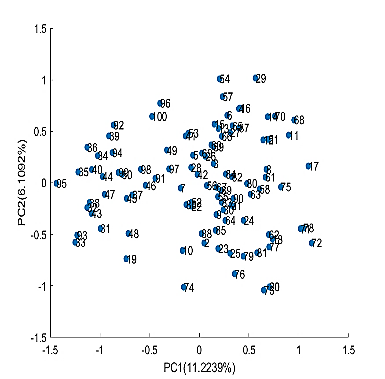

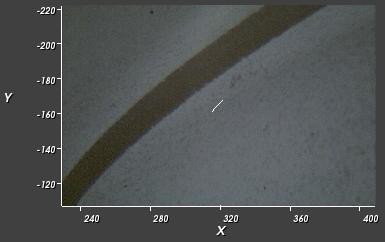

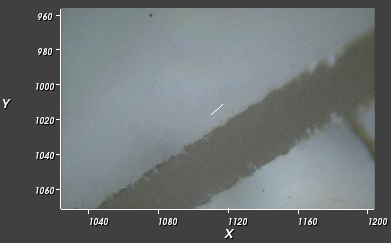

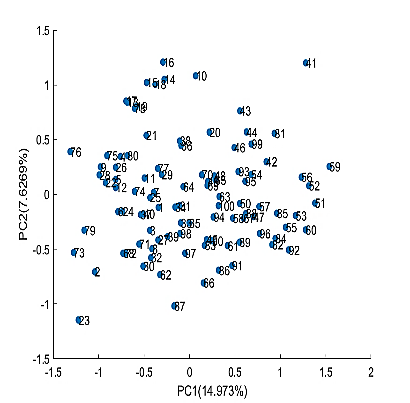

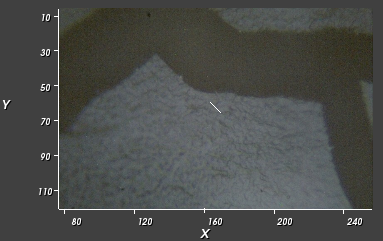

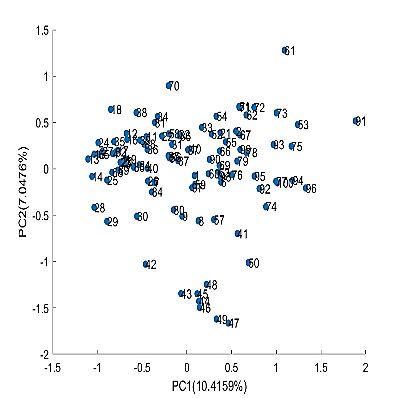


**(b)**

**(a)**

**Figure S1.** Representative images of the regions on the sample from where the line map was taken with corresponding sample PCA from both the (a) healthy and (b) GDM patients.

**Table S1**. Healthy patients’ clinical information including maternal age, gestational age at delivery (GA), body mass index (BMI), number of pregnancies (gravida), number of births (parity), number of pregnancy losses, blood sugar levels, and comorbidities.

| **Healthy group** | **Age (years)** | **BMI (Kg/m^2^)** | **GA (weeks)** | **Gravida** | **Parity** | **Pregnancy loss** | **Blood sugar concentration (mg/dL)** | **Comorbidities** |
| --- | --- | --- | --- | --- | --- | --- | --- | --- |
| 1 | 33 | 30.3 | 39.6 | 2 | 1 | 0 | 102 | None |
| 2 | 35 | 21.5 | 39.6 | 3 | 2 | 0 | 102 | None |
| 3 | 32 | 23.8 | 41.3 | 2 | 1 | 0 | 114 | None |
| 4 | 33 | 18.0 | 39.3 | 8 | 4 | 3 | 121 | None |
| 5 | 41 | 53.0 | 39.0 | 3 | 2 | 0 | 114 | Chronic hypertension, hypothyroidism, PCOS |
| 6 | 36 | 21.8 | 39.9 | 3 | 2 | 0 | 132 | None |
| 7 | 34 | 27.4 | 39.4 | 2 | 1 | 0 | 124 | None |
| 8 | 35 | 27.0 | 39.0 | 5 | 3 | 1 | 113 | None |
| 9 | 40 | 25.0 | 39.0 | 3 | 1 | 1 | 112 | None |
| 10 | 35 | 30.5 | 39.1 | 2 | 1 | 0 | 130 | None |
| 11 | 25 | 35.4 | 38.4 | 2 | 1 | 0 | 111 | None |
| 12 | 33 | 18.9 | 40.9 | 1 | 0 | 0 | 81 | Hypothyroidism |
| 13 | 30 | 22.7 | 37.1 | 1 | 0 | 0 | 110 | Gestational hypertension |
| 14 | 31 | 18.9 | 39.0 | 2 | 1 | 0 | 97 | None |
| 15 | 36 | 34.4 | 37.0 | 2 | 1 | 0 | 110 | Von Willebrand's disease, chronic hypertension |
| 16 | 23 | 36.4 | 38.1 | 3 | 2 | 0 | 110 | None |
| 17 | 19 | 21.6 | 39.6 | 1 | 0 | 0 | 145 | None |
| 18 | 29 | 24.1 | 39.0 | 4 | 2 | 1 | 101 | None |
| 19 | 36 | 21.1 | 38.0 | 1 | 0 | 0 | 135 | None |
| 20 | 28 | 26.5 | 34.4 | 3 | 1 | 1 | 117 | Severe preeclampsia, induced early due to PE |
| 21 | 25 | 22.2 | 37.3 | 6 | 2 | 2 | 72 | None |
| 22 | 33 | 22.2 | 40.3 | 2 | 1 | 0 | 110 | None |
| 23 | 38 | 21.9 | 39.7 | 3 | 1 | 1 | 85 | None |
| 24 | 30 | 51.3 | 32.0 | 5 | 2 | 2 | 120 | Early parturition due to decreased fetal movement |
| 25 | 31 | 33.6 | 39.9 | 3 | 2 | 1 | 112 | None |
| 26 | 37 | 27.1 | 39.1 | 2 | 1 | 0 | 136 | None |
| 27 | 33 | 32.9 | 38.1 | 2 | 0 | 1 | 141 | None |
| 28 | 25 | 45.2 | 38.6 | 1 | 0 | 0 | 137 | None |
| 29 | 31 | 59.6 | 39.1 | 1 | 0 | 0 | 172 | None |
| 30 | 33 | 23.2 | 34.7 | 1 | 0 | 0 | 135 | None |
| 31 | 31 | 31.2 | 39.1 | 2 | 1 | 0 | 125 | None |
| 32 | 29 | 34.9 | 39.1 | 1 | 0 | 0 | 87 | None |
| 33 | 34 | 23.7 | 39.3 | 3 | 2 | 0 | 118 | None |
| 34 | 29 | 37.7 | 39.3 | 2 | 1 | 0 | N/A | Chronic Hypertension, hypothyroidism |

**Table S2**. GDM patient's clinical information including maternal age, gestational age at delivery, body mass index (BMI), number of pregnancies (gravida), number of births (parity), number of pregnancy losses, blood sugar levels, and comorbidities

| **GDM group** | **Age (years)** | **BMI (Kg/m^2^)** | **GA (weeks)** | **Gravida** | **Parity** | **Pregnancy loss** | **Blood sugar concentration (mg/dL)** | **Comorbidities** |
| --- | --- | --- | --- | --- | --- | --- | --- | --- |
| 1 | 27 | 29.9 | 38.7 | 2 | 1 | 0 | 236 | None |
| 2 | 33 | 24.2 | 37.9 | 6 | 2 | 3 | 180 | None |
| 3 | 43 | 41.0 | 39.0 | 4 | 1 | 1 | 212 | None |
| 4 | 37 | 24.5 | 40.0 | 1 | 0 | 0 | 147 | None |
| 5 | 37 | 39.4 | 38.1 | 5 | 1 | 2 | 232 | None |
| 6 | 34 | 35.2 | 39.1 | 3 | 1 | 1 | 167 | None |
| 7 | 36 | 25.4 | 39.3 | 3 | 2 | 1 | 199 | None |
| 8 | 28 | 53.9 | 39.6 | 3 | 1 | 1 | 150 | None |
| 9 | 32 | 24.1 | 39.0 | 1 | 0 | 0 | 208 | None |
| 10 | 33 | 40.8 | 40.9 | 6 | 5 | 0 | 183 | None |
| 11 | 32 | 36.6 | 39.0 | 3 | 1 | 1 | 194 | None |
| 12 | 30 | 46.6 | 40.6 | 4 | 3 | 0 | 144 | None |
| 13 | 32 | 32.8 | 39.1 | 7 | 6 | 0 | 135 | None |
| 14 | 30 | 36.4 | 39.0 | 3 | 1 | 1 | 159 | None |
| 15 | 29 | 29.5 | 33.4 | 2 | 0 | 0 | 169 | None |
| 16 | 27 | 25.5 | 37.3 | 4 | 3 | 0 | 218 | None |
| 17 | 25 | 32.9 | 39.9 | 1 | 0 | 0 | 173 | None |
| 18 | 30 | 30.3 | 37.0 | 1 | 0 | 0 | 181 | None |
| 19 | 28 | 20.7 | 39.1 | 1 | 0 | 0 | 148 | None |
| 20 | 29 | 24.4 | 39.1 | 1 | 0 | 0 | 199 | None |
| 21 | 36 | 21.1 | 38.7 | 3 | 2 | 0 | N/A | None |
| 22 | 34 | 37.6 | 39.4 | 2 | 1 | 0 | N/A | None |
| 23 | 28 | 21.3 | 39.1 | 1 | 0 | 0 | N/A | None |
| 24 | 26 | 32.0 | 39.4 | 2 | 1 | 0 | 178 | None |
| 25 | 39 | 38.0 | 39.1 | 2 | 0 | 1 | 167 | None |
| 26 | 35 | 23.3 | 38.7 | 1 | 0 | 0 | 187 | None |
| 27 | 25 | 30.6 | 39.1 | 2 | 1 | 0 | 189 | None |
| 28 | 26 | 38.3 | 37.4 | 2 | 1 | 0 | 238 | None |
| 29 | 34 | 37.1 | 37.3 | 3 | 2 | 0 | 217 | None |
| 30 | 51 | 30.6 | 39.0 | 4 | 1 | 1 | 237 | None |
| 31 | 31 | 26.6 | 39.1 | 7 | 3 | 3 | 188 | None |
| 32 | 37 | 38.0 | 40.9 | 2 | 0 | 1 | 149 | Hypothyroidism |
| 33 | 31 | 23.4 | 38.4 | 1 | 0 | 0 | 189 | None |
| 34 | 22 | 35.1 | 39.1 | 1 | 0 | 0 | 176 | None |

**Figure S2**. Raman spectra of (a) all healthy and (b) all GDM patient samples included in this study.

**(a)**

**(b)**


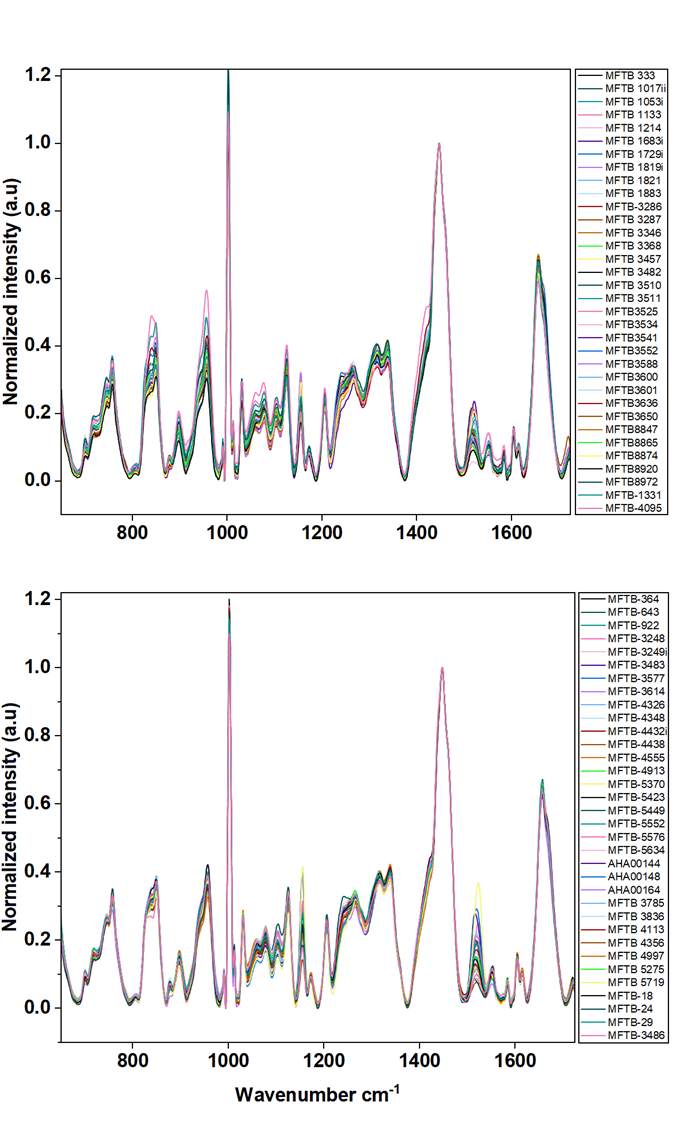


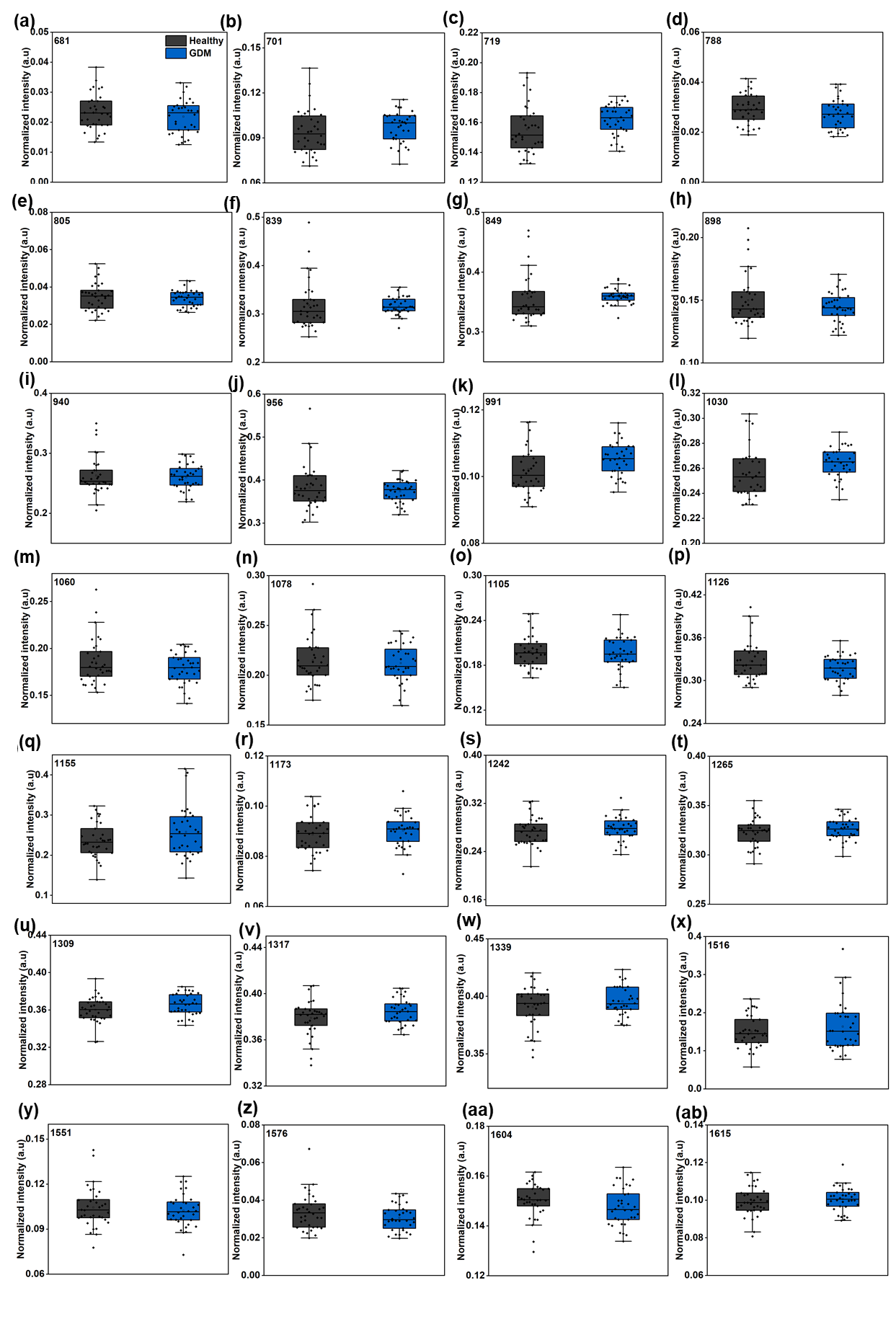


**Figure S3**. Representation of Raman metabolites as box plots that distinguish GDM **(blue)** from healthy **(black)** patients. (a) methionine (b) cholesterol, (c) phosphatidylcholine and sphingomyelin, (d,e) DNA/RNA, (f) proline, (g) sugars (glucose/glycerol), (h) glycine, (i) citric/succinic acid (j) myristic acid, (k) arginine, (l) phenylalanine, (m,n) lipids, (o) mannose, (p) glucose, (q) carotenoids, (r) saturated long-chain fatty acids, (s) amide III, (t) unsaturated lipids and fatty acids (u) triglycerides (v) histidine, (w) threonine, (x) carotenoids, (y) tryptophan, (z) DNA & NADH, (aa) phenylalanine, (ab) tyrosine. Here, the *p value>0.05* (non-significant) for all metabolites represented.

**Table S3**. List of key metabolites in association with the major pathways identified from MS KEGG pathway enrichment analysis.

| **Pathways** | **Total compounds** | **Hits** | **LC-MS Metabolites** |
| --- | --- | --- | --- |
| Starch and sucrose metabolism | 18 | 4 | Cellobiose, D-Fructose, Sucrose, D-Glucose |
| Glyoxylate and dicarboxylate metabolism | 32 | 7 | cis-Aconitic acid, Glycolic acid; Citric acid L-Serine; Glycine, L-Glutamic acid Glyceric acid |
| Fatty acid elongation | 38 | 1 | Palmitic acid |
| Fatty acid degradation | 39 | 1 | Palmitic acid |
| Fructose and mannose metabolism | 20 | 3 | Sorbitol, D-Fructose, D-Mannose |
| Glycerolipid metabolism | 16 | 1 | Glyceric acid |
| Amino sugar and nucleotide sugar metabolism | 37 | 3 | D-Galactose,D-Mannose, D-Fructose |
| Galactose metabolism | 27 | 7 | Sucrose, D-Galactose, D-Glucose, D-Fructose, D-Mannose,Sorbitol, myo-Inositol |
| Valine, leucine and isoleucine degradation | 40 | 3 | L-Valine,L-Leucine, Methylmalonic acid |
| Glycolysis / Gluconeogenesis | 26 | 2 | L-Lactic acid, Beta-D-Glucose |
| Biosynthesis of unsaturated fatty acids | 36 | 6 | (4Z,7Z,10Z,13Z,16Z,19Z)-Docosahexaenoyl-CoA, Palmitic acid, Stearic acid; Arachidic acid, Oleic acid, Linoleic acid, Arachidonic acid |
| Nicotinate and nicotinamide metabolism | 15 | 1 | L-Aspartic acid |
| Phenylalanine, tyrosine and tryptophan biosynthesis | 4 | 3 | Phenylpyruvic acid, L-Phenylalanine, L-Tyrosine |
| Phenylalanine metabolism | 10 | 3 | L-Phenylalanine, Phenylpyruvic acid, L-Tyrosine |
| Histidine metabolism | 16 | 3 | L-Glutamic acid, L-Histidine, L-Aspartic acid |
| Butanoate metabolism | 15 | 4 | L-Glutamic acid,Butyric acid, Oxoglutaric acid, Succinic acid |
| D-Glutamine and D-glutamate metabolism | 6 | 2 | L-Glutamic acid,Oxoglutaric acid |
| Selenocompound metabolism | 20 | 4 | Phosphoroselenoic acid, Selenate, Methylselenic acid,Methylselenopyruvate |
| Steroid hormone biosynthesis | 85 | 4 | Cholesterol,Progesterone, Tetrahydrocorticosterone,Cholesterol sulfate |
| Linoleic acid metabolism | 5 | 2 | Linoleic acid,13-L-Hydroperoxylinoleic acid |
| Nitrogen metabolism | 6 | 1 | L-Glutamic acid |
| Glycosphingolipid biosynthesis - lacto and neolacto series | 47 | 1 | Lactosylceramide (d18:1/12:0) |
| Glycosphingolipid biosynthesis - globo and isoglobo series | 15 | 1 | Lactosylceramide (d18:1/12:0) |
| Glycosphingolipid biosynthesis - ganglio series | 20 | 1 | Lactosylceramide (d18:1/12:0) |
| Alanine, aspartate and glutamate metabolism | 28 | 6 | L-Aspartic acid, L-Glutamic acid, Citric acid, Fumaric acid, Succinic acid, Oxoglutaric acid |
| Citrate cycle (TCA cycle) | 20 | 5 | Oxoglutaric acid,Succinic acid, cis-Aconitic acid, Citric acid, Fumaric acid |
| Ascorbate and aldarate metabolism | 8 | 1 | myo-Inositol |
| Inositol phosphate metabolism | 30 | 1 | myo-Inositol |
| Sphingolipid metabolism | 21 | 4 | Sphinganine, L-Serine, Lactosylceramide (d18:1/12:0),Phytosphingosine |
| Vitamin B6 metabolism | 9 | 1 | 2-Oxo-3-hydroxy-4-phosphobutanoic acid |
| Pentose phosphate pathway | 22 | 2 | Gluconic acid, Glyceric acid |
| Fatty acid biosynthesis | 47 | 3 | Palmitic acid, Myristic acid, Dodecanoic acid |
| Arachidonic acid metabolism | 36 | 3 | Arachidonic acid, Prostaglandin G2, 11,14,15-THETA |
| alpha-Linolenic acid metabolism | 13 | 1 | Stearidonic acid |
| N-Glycan biosynthesis | 39 | 1 | N-Acetyl-D-glucosaminyldiphosphodolichol |

**Figure S4.** Numeric Pearson correlation heatmap between RS peak values (unit = cm^-1^) and normalized clinical data for healthy (n=34) and GDM (n=34) patients. Correlation values are highlighted as one-to-one (=1.0), strongly positive (>0.51), moderately positive (>0.29), weakly positive (>0.0), weakly negative (< 0.0), moderately negative (<-0.29) and strongly negative (<-0.51).

**Figure S5.** (a) PCA scatter plots using clinical data to cluster healthy and GDM patient samples. (b) PCA scatter plot using RS data to cluster healthy and patient GDM samples. (c) PCA Loading Plot showing contribution of Raman spectroscopy peaks to principal components.

**(a)**

**(b)**

**(c)**


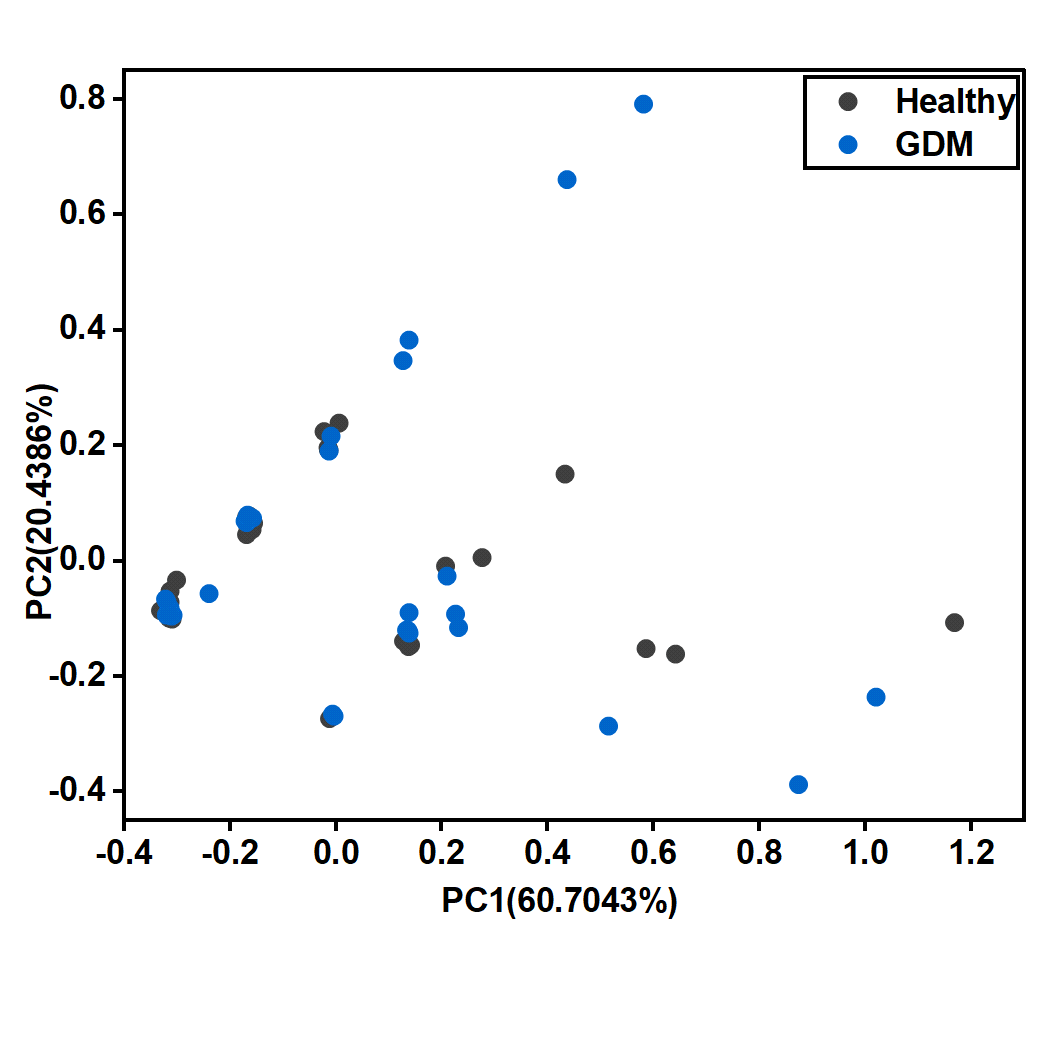

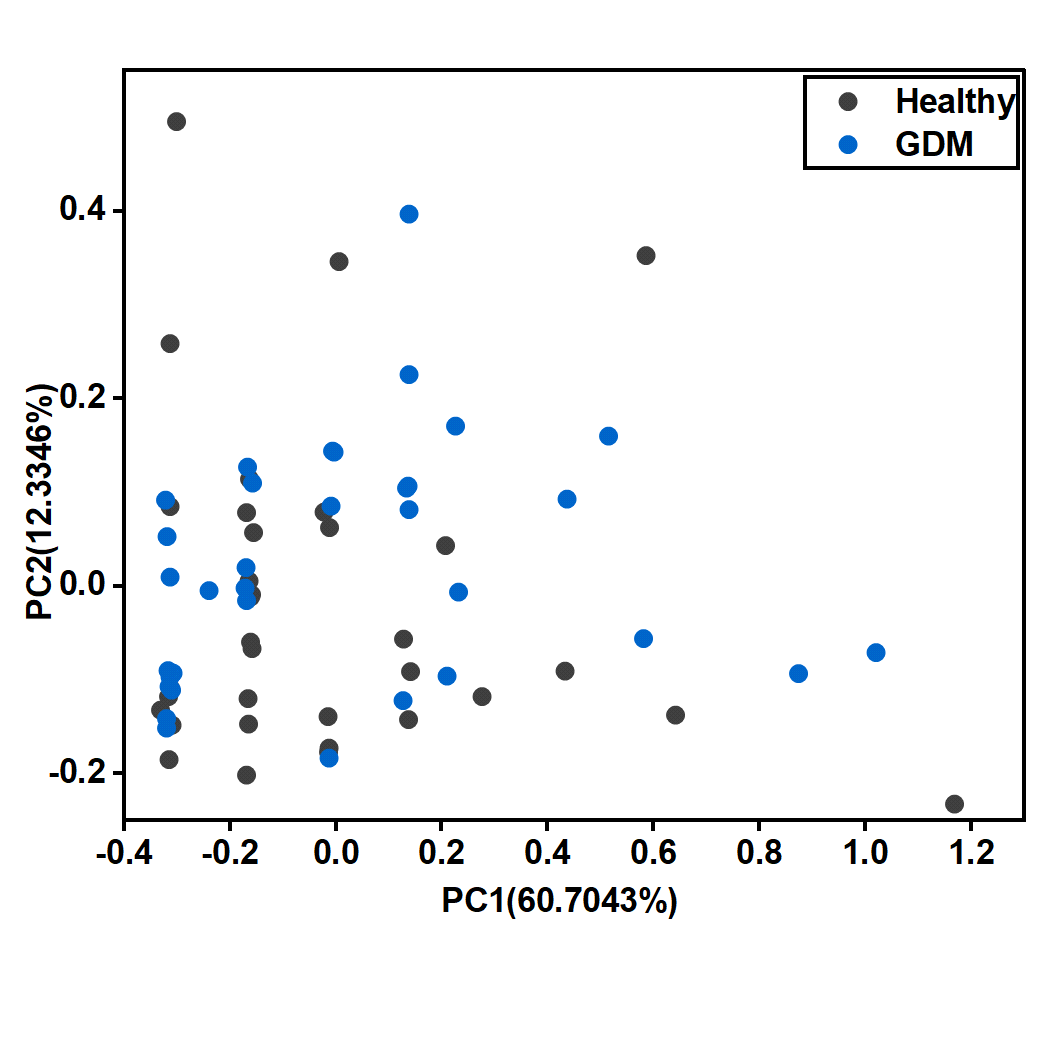

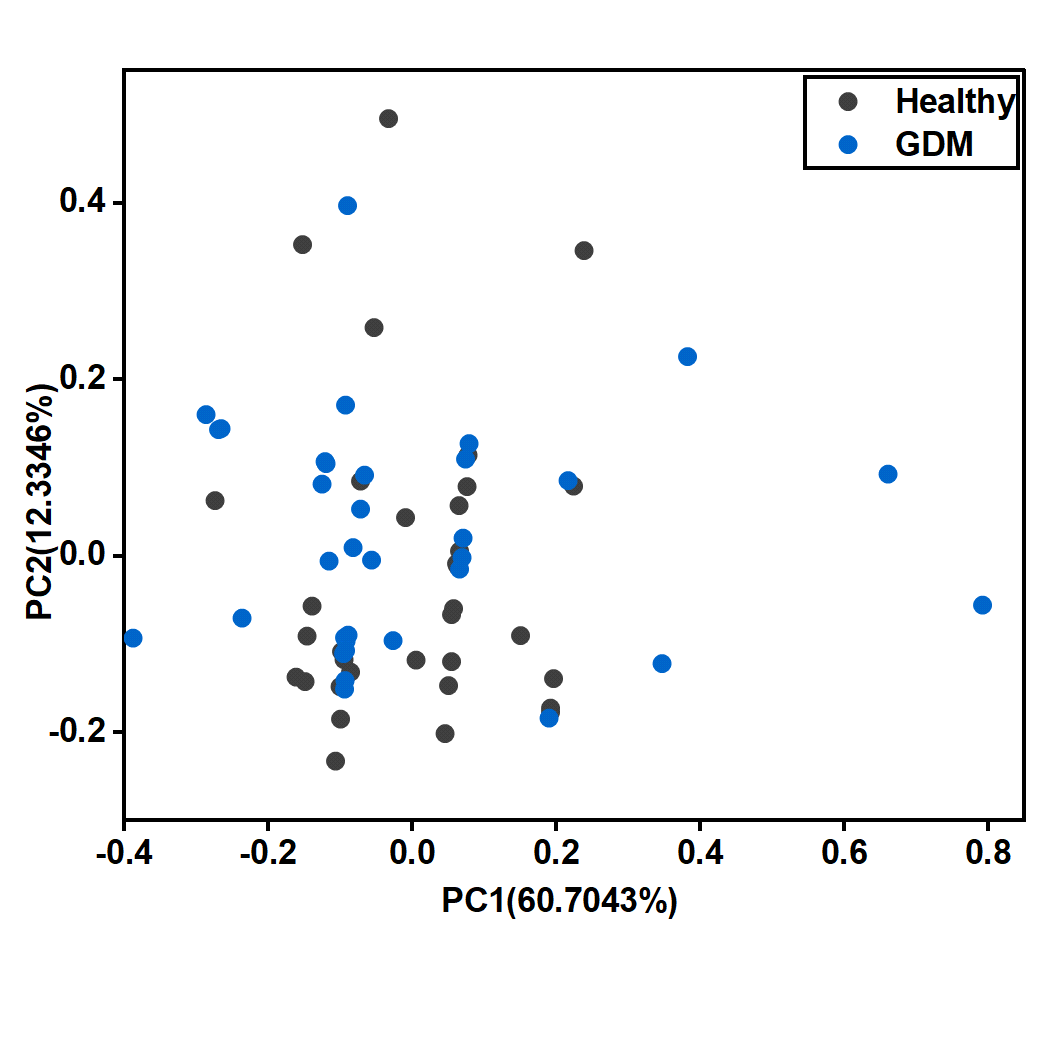

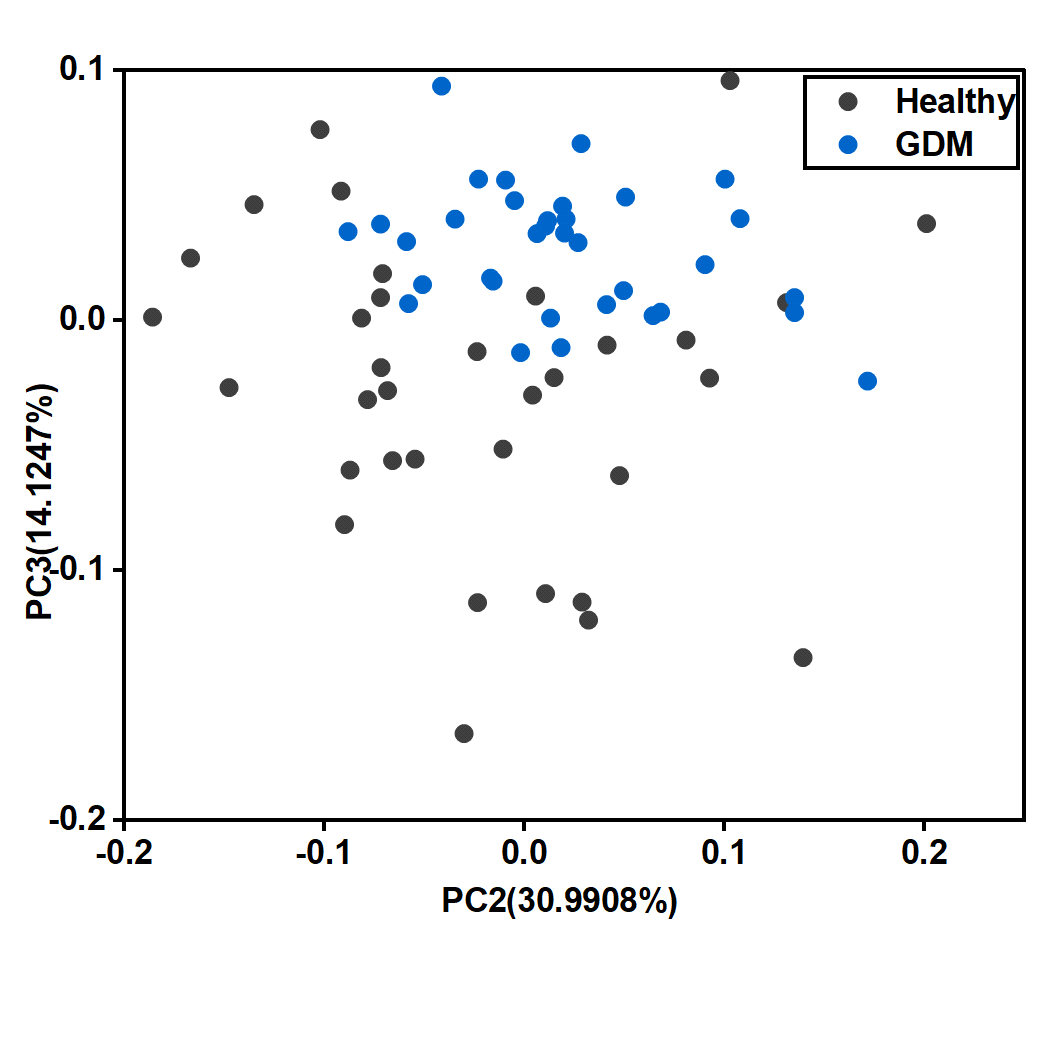

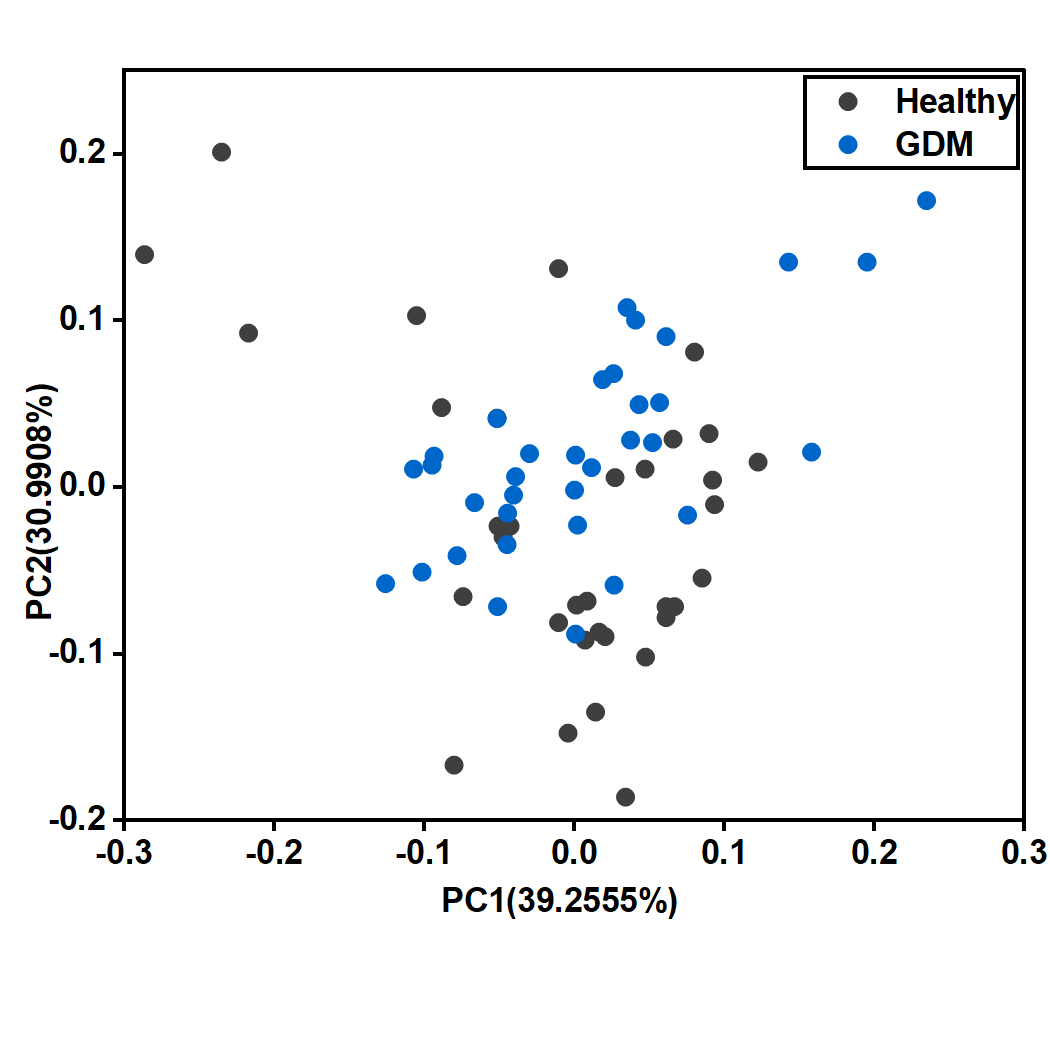

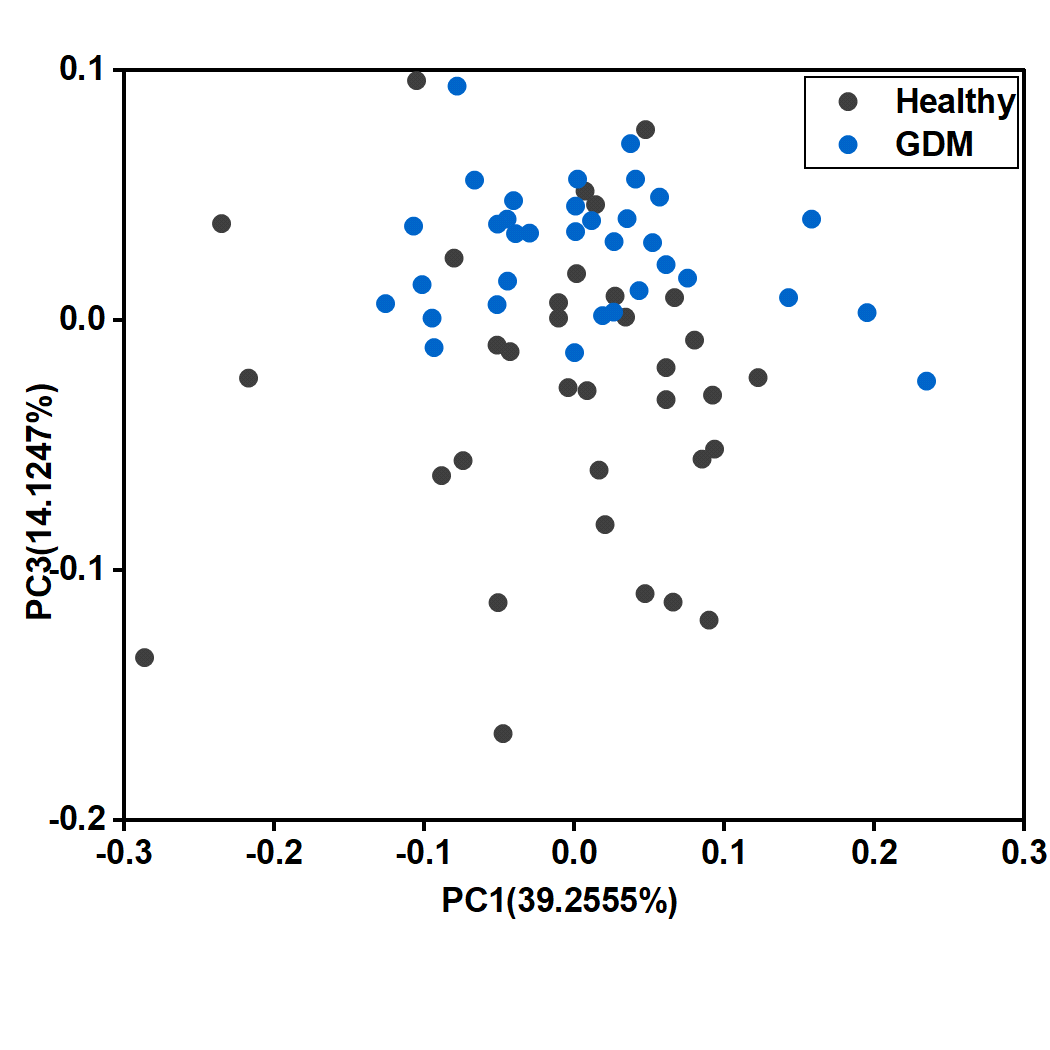


**Table S4.** Truncated feature list used for SVM classification.

| **Wavenumber (cm^-1^)** | **Metabolic assignment** |
| --- | --- |
| 681 | Methionine |
| 701 | Cholesterol |
| 719 | Phosphatidylcholine (PC) /sphingomyelin |
| 745 | Thymine (DNA bases) |
| 757 | Tryptophan |
| 828 | Tyrosine |
| 839 | Proline |
| 849 | Sugars (Glucose, Glycerol) |
| 878 | Glutamic acid |
| 898 | Glycine |
| 940 | Citric acid, Succinic acid |
| 956 | Myristic Acid |
| 991 | Arginine |
| 1002 | Phenylalanine |
| 1013 | Carbohydrates |
| 1060 | Lipids |
| 1078 | Lipids |
| 1105 | Mannose |
| 1126 | Glucose |
| 1155 | Carotenoids |
| 1173 | Saturated fatty acids |
| 1206 | Amino acids |
| 1242 | Amide III |
| 1265 | Unsaturated lipids, fatty acids |
| 1309 | Triglycerides |
| 1317 | Histidine |
| 1339 | Threonine |
| 1576 | DNA & NADH |
| 1657 | Unsaturated lipids, PC, phosphatidylethanolamine |
| 1670 | Amide I |

**Table S5.** Summary of SVM model hyperparameter optimization and performance. Error in cross-validation accuracy given as 1 standard deviation.

| Feature set | Validation method | Kernel | Degree | Regularization  (C-value) | Cross-validation accuracy |
| --- | --- | --- | --- | --- | --- |
| Clinical | 5-fold | Linear | N/A | 4 | 51.4% ± 5.3% |
| RS Peaks | 5-fold | Linear | N/A | 7 | 92.6% ± 4.5% |
| ELISA | 5-fold | Linear | N/A | 47 | 81.6% ± 3.9% |
| Clinical | 5-fold | Poly | 6 | 80 | 63.1% ± 9.3% |
| RS Peaks | 5-fold | Poly | 2 | 2 | 95.5% ± 3.7% |
| ELISA | 5-fold | Poly | 2 | 1 | 81.5% ± 8.0% |
| Clinical | 5-fold | RBF | N/A | 21 | 61.7% ± 9.6% |
| RS Peaks | 5-fold | RBF | N/A | 3 | 91.2% ± 2.8% |
| ELISA | 5-fold | RBF | N/A | 25 | 83.1% ± 7.3% |
| Clinical | LOOCV | Linear | N/A | 2 | 48.5% ± 50.0% |
| RS Peaks | LOOCV | Linear | N/A | 80 | 92.6% ± 26.1% |
| ELISA | LOOCV | Linear | N/A | 7 | 81.7% ± 38.7% |
| Clinical | LOOCV | Poly | 6 | 83 | 64.7% ± 47.8% |
| RS Peaks | LOOCV | Poly | 2 | 2 | 95.6% ± 20.5% |
| ELISA | LOOCV | Poly | 1 | 2 | 80.3% ± 39.8% |
| Clinical | LOOCV | RBF | N/A | 20 | 60.3% ± 48.9% |
| RS Peaks | LOOCV | RBF | N/A | 2 | 91.2% ± 28.4% |
| ELISA | LOOCV | RBF | N/A | 32 | 85.9% ± 34.8% |


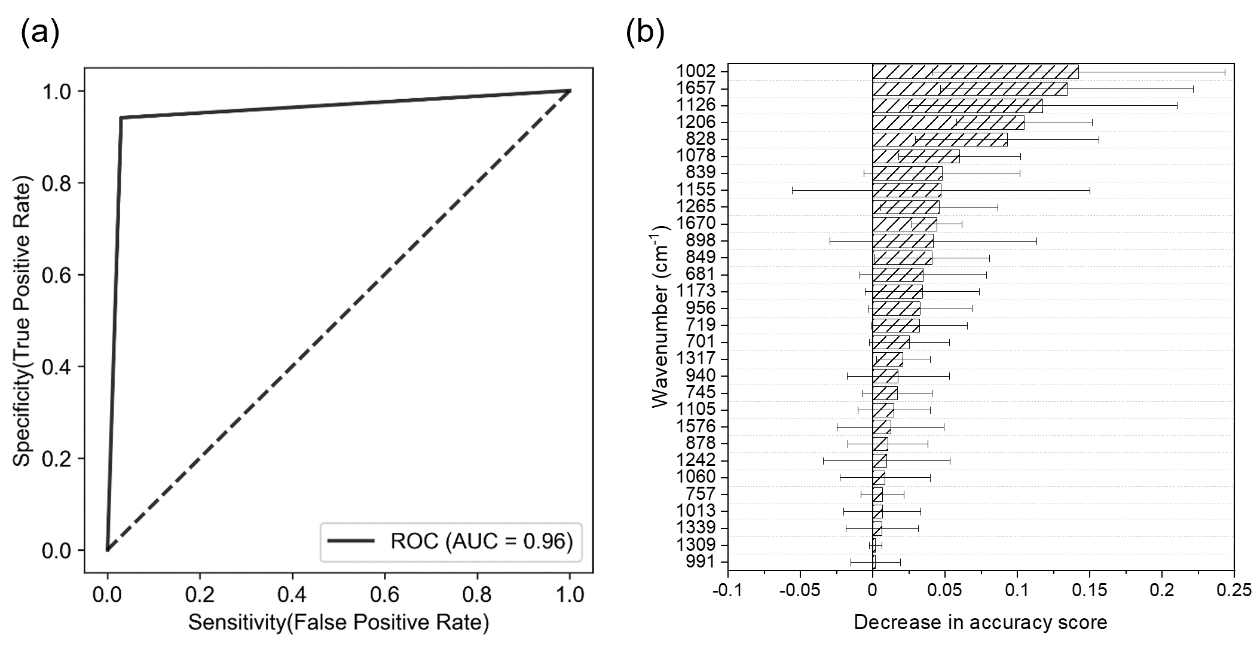


**Figure S6.** Performance of optimized polynomial kernel SVM model trained on RS peak data to differentiate (n=34) GDM and (n=34) healthy samples. (a) AUC-ROC curve for binary classification analysis using leave-one-out cross-validation. (b) Permutation importance analysis of (n=68) LOOCV test-train folds with 1 standard deviation error in decrease in accuracy from feature permutation.


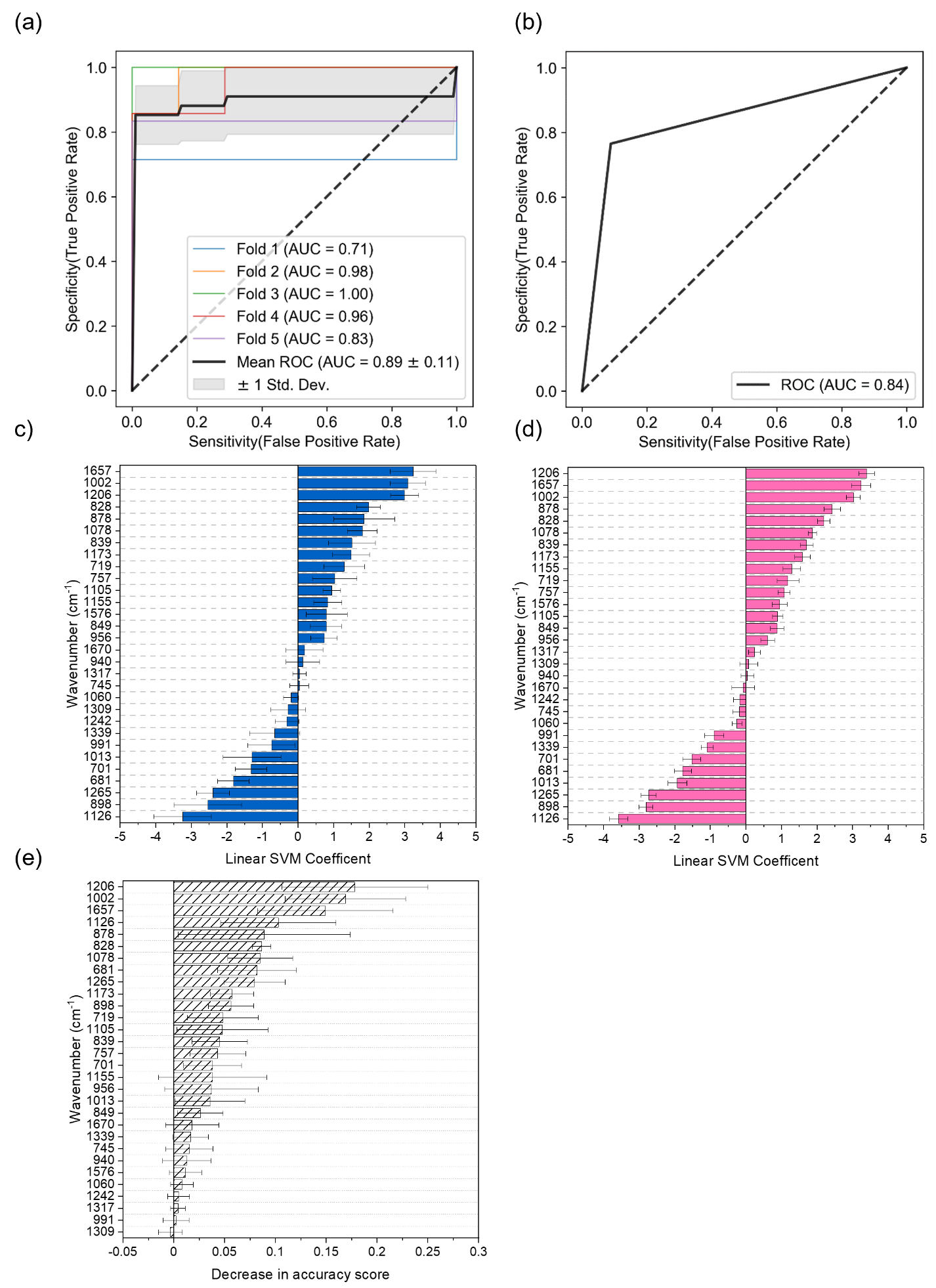


**Figure S7.** Performance of optimized linear kernel SVM model trained on RS peak data to differentiate (n=34) GDM and (n=34) healthy samples. (a) AUC-ROC curves for binary classification analysis using 5-fold cross-validation. (b) AUC-ROC curve for binary classification analysis using leave-one-out cross-validation. (c) Mean linear SVM coefficients of 5-fold cross-validation test-train folds with 1 standard deviation error. (d) Mean linear SVM coefficients of leave-one-out cross-validation test-train folds with 1 standard deviation error. (e) Mean linear SVM coefficients of 5-fold cross-validation test-train folds with 1 standard deviation error.


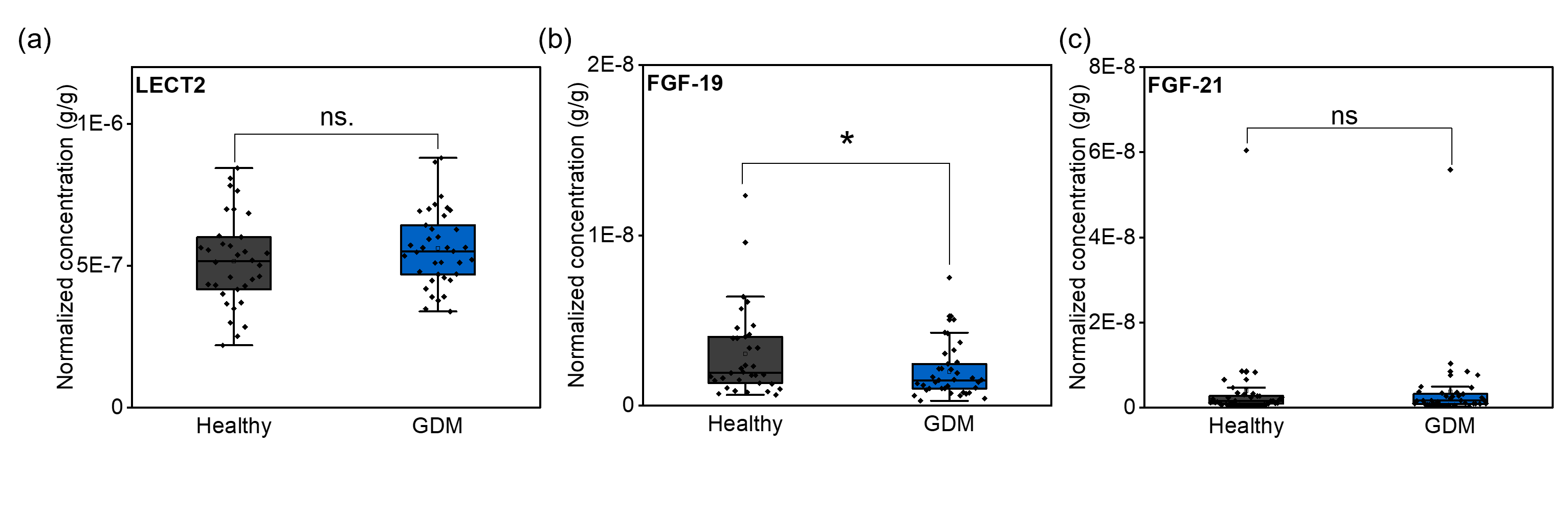


**Figure S8.** Protein expression for (a) LECT2, (b) FGF-19 and (c) FGF-21 normalized to total protein content for n=37 GDM and n=34 healthy patients.


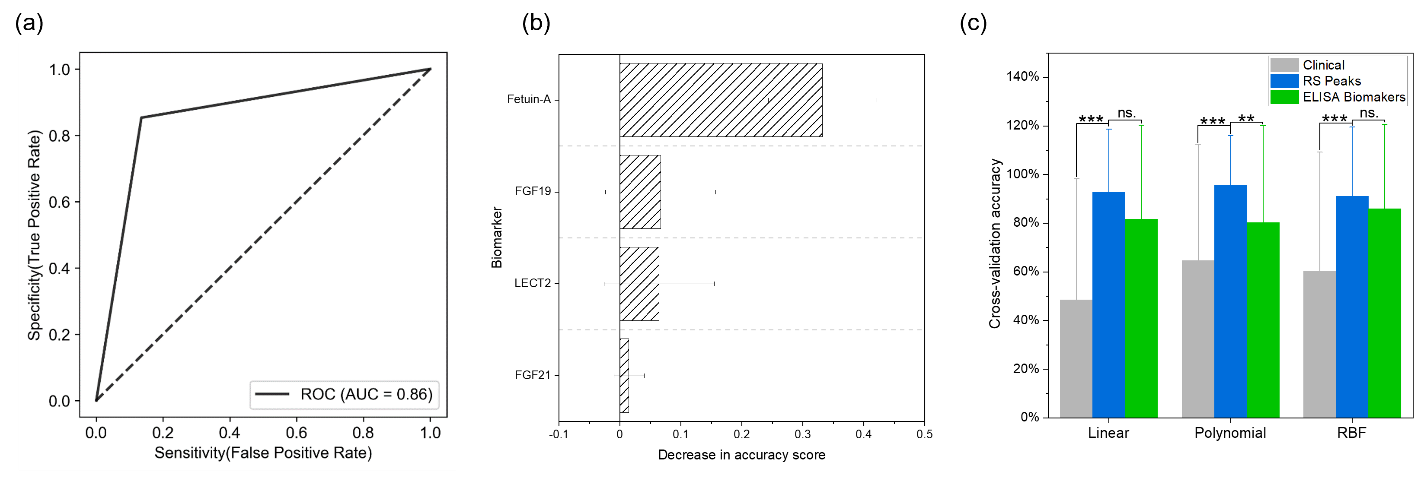


**Figure S9.** (a-b) Performance of optimized radial basis function kernel SVM model trained on ELISA biomarker data to differentiate (n=37) GDM and (n=34) healthy samples. (a) AUC-ROC curve for binary classification analysis using leave-one-out cross-validation. (b) Permutation importance analysis of (n=68) LOOCV test-train folds with 1 standard deviation error in decrease in accuracy from feature permutation. (c) Leave-one-out cross-validation accuracies for SVM binary classification models trained on clinical, RS peak and ELISA biomarker datasets using the linear, polynomial, and radial basis function kernels. Error-bars indicate 1 standard deviation.


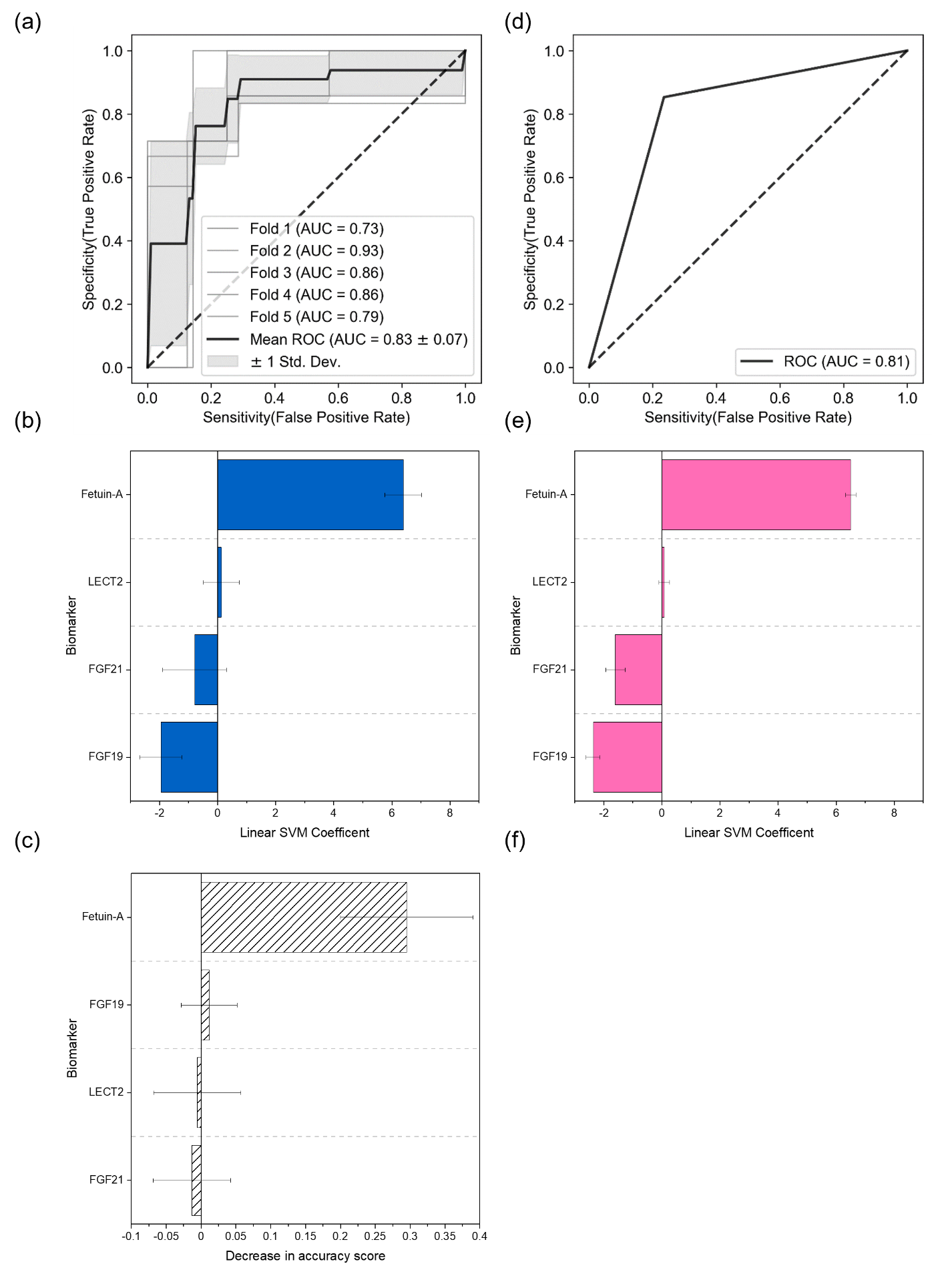


**Figure S10.** Performance of optimized linear kernel SVM model trained on ELISA biomarker data to differentiate (n=37) GDM and (n=34) healthy samples. (a) AUC-ROC curves for binary classification analysis using 5-fold cross-validation. (b) AUC-ROC curve for binary classification analysis using leave-one-out cross-validation. (c) Mean linear SVM coefficients of 5-fold cross-validation test-train folds with 1 standard deviation error. (d) Mean linear SVM coefficients of leave-one-out cross-validation test-train folds with 1 standard deviation error. (e) Mean linear SVM coefficients of 5-fold cross-validation test-train folds with 1 standard deviation error.


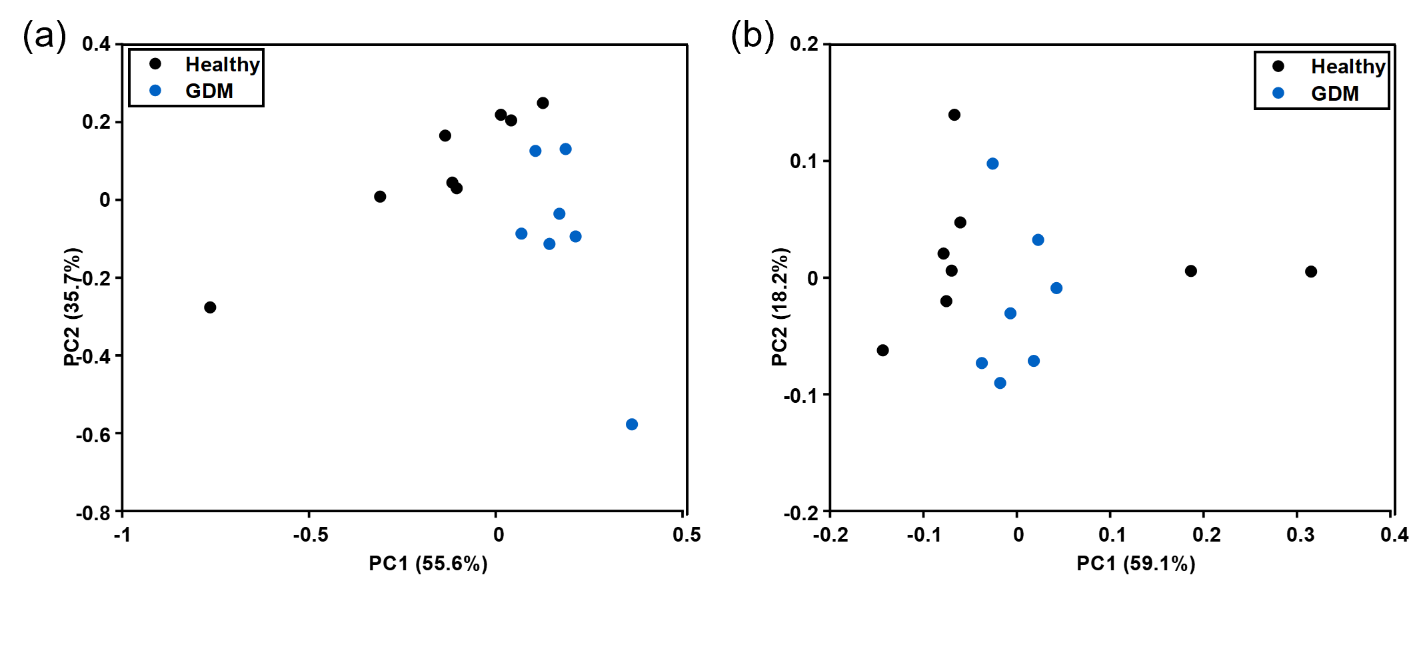


**Figure S11.** (a) PCA analysis plot comparing PC1 and PC2 using ELISA data for n=8 healthy and n=7 GDM samples. (b) PCA analysis plot comparing PC1 and PC2 using RS data for n=8 healthy and n=7 GDM samples

**Figure S12.** Numeric Pearson correlation heatmap between RS peak values (unit = cm^-1^) and normalized protein biomarker data for (n=8) healthy and (n=7) GDM patient subset. Correlation values are highlighted as one-to-one (=1.0), strongly positive (>0.51), moderately positive (>0.29), weakly positive (>0.0), weakly negative (< 0.0), moderately negative (<-0.29) and strongly negative (<-0.51).


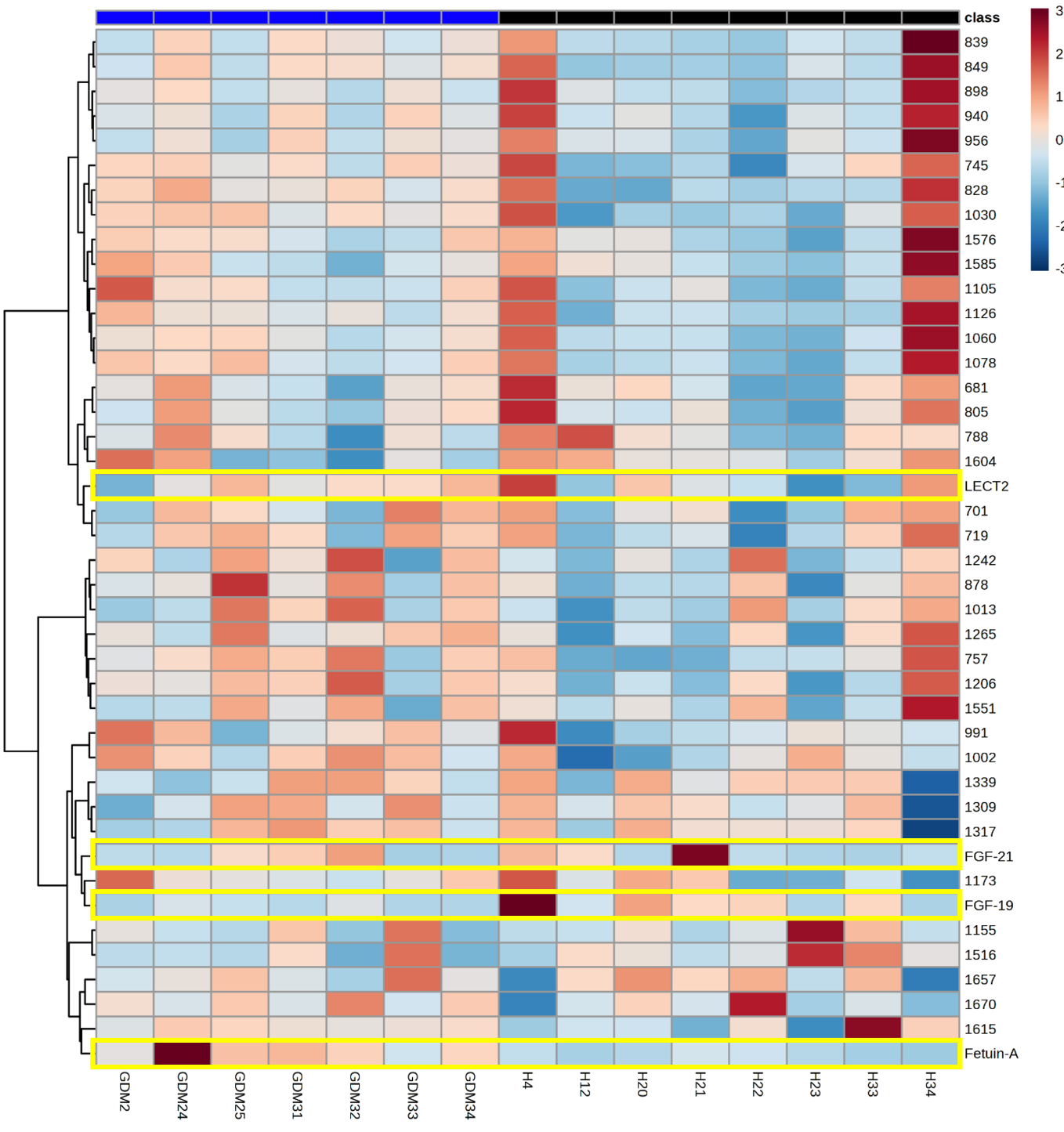


**Figure S13.** Feature intensity heatmap for the (n=7) GDM and (n=8) healthy patient samples used for RS peak and biomarker correlation analysis. Features are clustered based on Euclidian distance. Protein biomarkers are highlighted.
